# Plains Zebras Prioritize Foraging Without Sacrificing Social Bonds During a Severe Drought

**DOI:** 10.1002/ece3.70632

**Published:** 2025-01-08

**Authors:** Severine B. S. W. Hex, Erin S. Isbilen, Daniel I. Rubenstein

**Affiliations:** ^1^ Department of Ecology and Evolutionary Biology Princeton University Princeton New Jersey USA; ^2^ Child Study Center Yale University, School of Medicine New Haven Connecticut USA

**Keywords:** climate change, *Equus quagga*, multimodal communication, social networks

## Abstract

Anthropogenically induced climate change has significantly increased the frequency of acute weather events, such as drought. As human activities amplify environmental stresses, animals may be forced to prioritize survival over behaviors less crucial to immediate fitness, such as socializing. Yet, social bonds may also enable individuals to weather the deleterious effects of environmental conditions. We investigated how the highly social plains zebra ( *Equus quagga* ) modify their activity budgets, social networks, and multimodal communication during a drought. Although animals prioritized feeding and the number of social interactions dramatically decreased in the late drought period, social associations remained robust. We observed age/sex class‐specific changes in social behavior, reflecting the nutritional needs and social niche of each individual. Stallions devoted more time to greeting behaviors, which could mitigate harassment by bachelor males and facilitate grazing time for the females of the harem. Juveniles significantly increased time spent active socializing, despite mothers showing the greatest decrease in the number of social interactions. Instead, unrelated, nonlactating females served as social partners, accommodating both juveniles' social needs and lactating mothers' nutritive requirements. Using a network‐based representation of multimodal communication, we observed a decrease in the number of signals used during the drought. Individuals used less diverse multimodal combinations, particularly in the costly context of aggression. These findings illustrate how social roles and differential responses to acute environmental stress within stable social groups may contribute to species resilience, and how communication flexibly responds to facilitate both survival and sociality under harsh environmental conditions.

## Graphical abstract

We investigated how the highly social plains zebra ( *Equus quagga* ) modify their activity budgets, social networks, and multimodal communication during a drought. Although animals prioritized feeding and the number of social interactions dramatically decreased in the late drought period, social associations remained robust. Nuanced changes in social interactions were facilitated by decreases in the size and combinatorial flexibility of multimodal repertoires, particularly in the costly context of aggression.

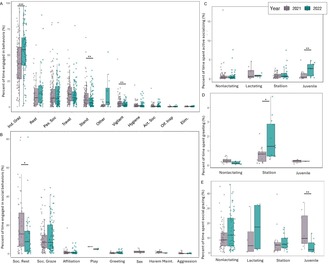

## 1 Introduction

The world is inherently variable, and animals have evolved a variety of physiological, life history, and behavioral adaptations to cope with fluctuations in the environment across time (Rymer, Pillay, and Schradin 2016; Varpe 2017; Rondeau and Raine 2024). Yet in recent years, global climate change has increased the frequency, intensity, and unpredictability of inhospitable climactic conditions (IPCC 2014; Buchholz et al. 2019). Drought, characterized by unusually low water availability, is a particular challenge, forcing organisms to endure concurrent hydric, nutritive, and thermal stress (Rymer, Pillay, and Schradin 2016; Menzel and Feldmeyer 2021). In the past century, the regional incidences of drought have increased 50% and are predicted to continue rising globally, particularly in arid and semi‐arid ecosystems (IPCC 2023). Climate change is an inexorable feature of the Anthropocene, and in response to shifting environmental conditions, animals will be increasingly forced to behaviorally respond. To predict species' resilience and inform conservation decisions, it is critical to understand not only evolved mechanisms that organisms can use to weather extreme climactic events, but also how behavioral flexibility can enable individuals to survive the escalating extremity of environmental stresses (Buchholz et al. 2019; Komdeur and Ma 2021; Moss and While 2021; McMillan et al. 2022; Blumstein, Hayes, and Pinter‐Wollman 2023).

One manner by which social animals can respond to environmental stress is by maximizing behaviors that enable them to acquire resources, retain moisture, and minimize heat stress, while reducing behaviors less crucial to immediate fitness, such as affiliation, agonism, vigilance, and reproduction (Ismail et al. 2011; Ramona, Clutton‐Brock, and Manser 2019). In some species, especially those which form fission–fusion societies, social networks become sparser in response to dry conditions and food scarcity to avoid competition (Waterman and Fenton 2000; Cunningham and Wronski 2011; Holekamp et al. 2012). However, there may also be forces compelling animals to maintain—or even strengthen—their social bonds in times of hardship (Cohen and Willis 1985; Beery and Kaufer 2015). Increasing evidence highlights how the number and especially the strength of social bonds impacts individual fitness, reproductive success, and survivorship in obligately social mammals (Silk, Alberts, and Altmann 2003; Silk et al. 2009, 2010; Cameron, Setsaas, and Linklater 2009; Ramp et al. 2010; Snyder‐Mackler et al. 2020). Cooperative social bonds may become particularly important in times of catastrophic disruptions to the social or physical environment (Foley, Pettorelli, and Foley 2008; Nuñez, Adelman, and Rubenstein 2015; Johnston, Lanham & Bull, 2020; Testard et al. 2021; Komdeur and Ma 2021). Nonetheless, relationships require time and energy to maintain, especially those between genetically unrelated individuals, and environmental stressors such as heightened temperatures and food unavailability may exacerbate these costs and destabilize associations (Moss and While 2021). Therefore, acute environmental challenges may impose constraints on the amount of time individuals can devote to bond formation and maintenance, forcing animals to navigate the crucial balance between self‐maintenance and sociality.

How an individual weighs this balance is likely influenced by a complex interplay of biotic and abiotic factors (Blumstein, Hayes, and Pinter‐Wollman 2023). Whether the resources a species relies upon are spatially and/or temporally patchy, monopolizable, and usurpable (contest competition) or relatively evenly distributed and shareable (scramble competition) will shape the competition dynamics that arise from social association (Nicholson 1954; Isbell and Young 2002). Further, physiological needs and social strategies are not homogenous across age/sex classes, and these differences may impact the responses a given individual can mount to environmental stress (Conradt, Clutton‐Brock, and Guinness 2000; Marchand et al. 2015; Marshall et al. 2016).

**FIGURE 1:**
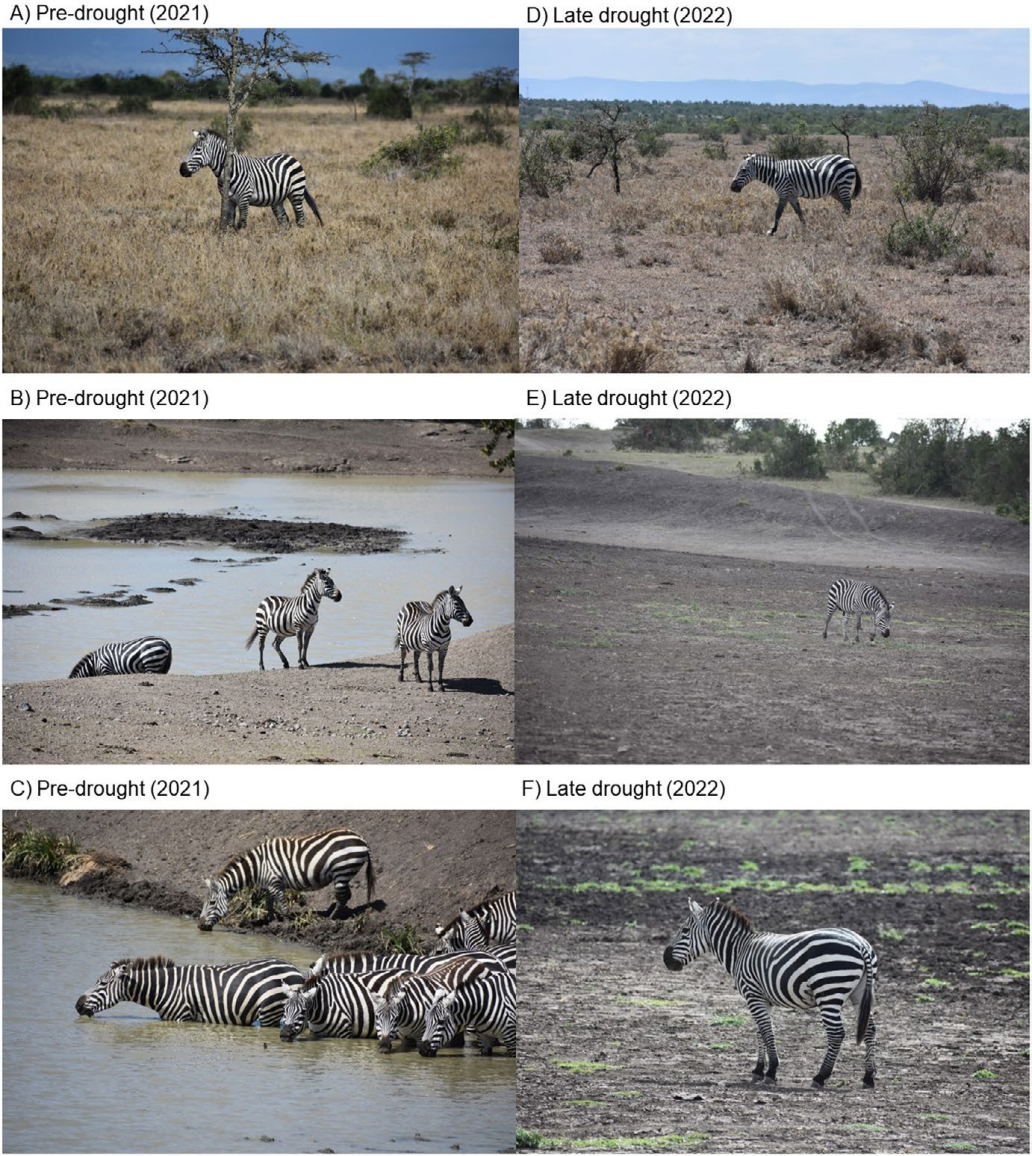
The difference in food and water availability during the pre‐ (2021) and late (2022) drought periods. In the pre‐drought, representing a typical dry season, grass was largely dry and of poor nutritive value, but still relatively abundant (A) and there was water in the dams throughout the conservancy (B, C). However, by the late drought period, overgrazing had reduced groundcover (D) and the same dams were completely dry (E, F).

For meaningful comparisons to be made before and after extreme weather events, studies must leverage longitudinal data on known individuals spanning periods of acute climactic stress. Between 2021 and 2022, the Laikipia‐Samburu ecosystem of Kenya experienced a complete failure of two consecutive rain seasons, resulting in an unusually severe drought with an extreme vegetation cover deficit (National Drought Management Authority 2022; Malonza 2022). This drought profoundly impacted wildlife mortality, claiming 512 wildebeest ( *Connochaetes taurinus* ), 205 African bush elephants ( *Loxodonta africana* ), and 2% of the critically endangered Grevy's zebra ( *Equus grevyi* ) population (Miwu et al. 2022) (Figure 1). Here, we examined the changes in activity budget, social behavior, and communication in a monitored wild population of plains zebras ( *Equus quagga* ) during this drought.

The core of plains zebra society is a stable unit called a “bonded true harem” (hereafter simply “harem”) (Hex, Tombak, and Rubenstein 2021), comprising a single reproductive stallion, 2–5 unrelated adult mares, and their dependent offspring (Tong, Shapiro, and Rubenstein 2015). The harem is a cooperative unit in which all members support groupmates from social and ecological threats; stallions offer protection to their mares from bachelor (i.e., unbonded, nonreproductive males) harassment and predators (Rubenstein and Nuñez 2009; Simpson, Rands, and Nicol 2012), while mares engage in mutual protection against male harassment (Hex et al. 2022) and joint vigilance (Klingel 1974). Plains zebras are less susceptible to droughts than other ungulates due to their physiology as hind gut fermenters, which allows them to extract nutrition even from low quality forage (Ogutu and Owen‐Smith 2003; Redfern et al. 2003). They typically dedicate 50%–80% of their daily activity budget to feeding (Clauss 2013) and have the flexibility to respond to environmental stress by increasing the amount of time they allocate to grazing. Therefore, this is a time‐limited system in which basic maintenance behaviors, especially grazing, must be balanced with the demonstrable benefits of maintaining strong social bonds.

Using the data spanning pre‐ (summer 2021) and late (summer 2022) drought periods, we investigated three questions. First, we asked how individuals modified their activity budgets and social behavior to the drought in accordance with their age/sex class. We predicted that zebras would shift their activity budgets to prioritize feeding and searching for food, to the detriment of active socializing (Ismail et al. 2011; Waterman and Fenton 2000). Second, we asked how individuals changed their patterns of intra‐harem social association in response to a drought‐altered resource landscape of low vegetation cover (Komdeur and Ma 2021; National Drought Management Authority 2022; Malonza 2022). We predicted four ways in which individuals could change their intra‐harem associations (Table 1). If resources became patchier on the landscape, intra‐harem social networks were predicted to become weaker and sparser as individuals spread out to reduce feeding competition and avoid aggression (Fattorini et al. 2023) (H_1_). However, if social bonds are too valuable to entirely dissolve, we predicted that individuals might only permit particularly close associates to graze in close proximity (Holekamp et al. 2012), resulting in harems showing greater heterogeneity in bond strength (H_2_). Alternatively, it is possible that weak bonds are important to maintain (Carter, Farine, and Wilkinson 2017; McFarland et al. 2017), while stronger bonds can tolerate a temporary reduction in investment. If this is the case, harems may become more homogenous in bond strength as the strongest bonds are reduced and weaker bonds are retained (H_3_). Finally, as grass is generally considered a permissive resource, it is possible that drought may not influence feeding competition, thus easing the tension between self‐maintenance and social bond maintenance. In this case, intra‐harem social networks and connectivity may remain relatively unchanged in response to the drought (H_4_).

**TABLE 1:** Hypotheses and predictions based upon how intra‐harem social networks and individual patterns of association might respond to the drought under different assumptions of resource distribution and resultant feeding competition.

Original social network		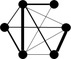
Hypothesis	Prediction	Representative network
Assumption: Drought leads to increased feeding competition		
H_1_: Social bonds are less important than self‐maintenance	Harem density: *decrease* Harem‐level CV: *decrease* Individual average SRI: *decrease* Individual CV: *decrease* Dyadic SRI: *decrease* Degree: *decrease*	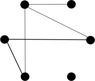
H_2_: Individuals tolerate only their strongest social bonds and eliminate or reduce weaker bonds	Harem density: *decrease* Harem‐level CV: *increase* Individual average SRI: *decrease* Individual CV: *increase* Degree: *decrease*	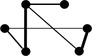
H_3_: Individuals reduce the time spent with the strongest associates to maintain the presence of weak bonds	Harem density: *no change* Harem‐level CV: *decrease* Individual average SRI: *decrease* Individual CV: *decrease* Degree: *no change*	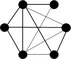
Assumption: Drought does not affect feeding competition		
H_4_: Individuals do not need to modify associations in response to drought	Harem density: *no change* Harem‐level CV: *no change* Individual average SRI: *no change* Individual CV: *no change* Dyadic SRI: *no change* Degree: *no change*	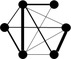

*Note:* We include cartoon social networks to illustrate how networks might respond under different hypotheses.

Abbreviations: CV, coefficient of variance of association strengths; Degree, number of connections an individual has; SRI, simple ratio index of association strength.

Third, we asked how individuals modified their communication to more effectively navigate their social environment during periods of environmental stress. Communication is an indispensable tool animals use to navigate their social environments, and how individuals communicate may also shift alongside altered social and environmental conditions. Previous work has shown that plains zebras possess a combinatorically complex, graded repertoire of visual, tactile, olfactory, and acoustic signals (Hex and Rubenstein 2024; Xie et al. 2024). We predicted that during the drought, the frequency of active social interactions would decrease, particularly in lactating females, who have the most intense water and food requirements (Trivers 1974; Hrdy 1999; Fite et al. 2005; Quinlan 2007; Fischhoff et al. 2007). Further, we predicted that structure of communication would become simpler and less combinatorically flexible to reduce the risks of costly misinterpretation or ambiguity, especially in more energetically expensive forms of interaction, like aggression.

Overall, our predictions were for plains zebras to modify their activity budgets to meet their self‐maintenance requirements at the expense of social behavior, while shifting their patterns of social behavior and communication to respond to the environmental stress of drought.

## 2 Materials and Methods

### 2.1 Ethics Statement

This study was conducted on a population of wild plains zebras living in Ol Pejeta Nature Conservancy (0°00 N, 36°56 E), in Laikipia, Kenya. All research was conducted with the permission of Ol Pejeta Nature Conservancy and the Kenyan Ministry of Wildlife (NACOSTI/P/21/11647) and was in compliance with the guidelines of the Princeton University Institutional Animal Care and Use Committee (1835F).

### 2.2 Study Animals

We focused our investigation on a sample of 21 focal harems with an average harem size of 5 members (2–13 individuals), comprising 109 individuals total (Table S1). The same harems were followed across both years, and the focal follow methodology used for collecting video recordings and association data were standardized across years. This population has been monitored continuously since 2008, and we have pedigrees of all focal individuals. We were able to individually identify zebras based on their unique stripes. Individuals were classified into one of four age/sex classes: stallion, nonlactating female, lactating female, and juvenile (Table S2). Juveniles were defined as pre‐dispersal individuals, including pre‐dispersed, sexually mature males as old as 4 years (*N* = 4 in 2021, *N* = 2 in 2022), who are nonreproductive and still operate in the juvenile social niche (Klingel 1965; personal observation). There was no significant difference in the average age of juveniles in 2021 (8.95 ± 8.8 months old) and 2022 (7.81 ± 5.41 months old).

### 2.3 Data Collection

Data collection occurred daily between July 13th and September 30th, 2021 (hereafter “pre‐drought”) and June 13th and August 12th, 2022 (hereafter “late drought”). Two focal follows were conducted each day by vehicle, the first occurring between 08:00 and 12:00 and the second between 12:30 and 16:30. A single harem was the subject of each observation period, and two harems were followed per day. At the beginning of each observation period, we recorded the presence or absence of members, including newly born foals, and scored the body condition of each individual using the plains zebra body condition scale, a qualitative measure of the overall body condition (Ginsberg 1988). Within each observation period, a single individual from the harem was randomly selected as the focal animal, to be followed for two consecutive 30 min blocks before a new focal individual was selected. The focal individual's behavior was continuously captured using a Canon Vixia HR R700 and a Sennheiser ME66 directional microphone in a Rode Blimp windshield and a Tascam Dr‐100MKII Linear PCM recorder sampling at 48 kHz. As it is challenging to extract meaningful behavior from video recordings taken while the vehicle is in motion, if individuals engaged in continuous travel for an extended duration (> 15 continuous minutes), we terminated the video recording and manually recorded the start and end time of travel for later integration into time budget calculations. Between each 30‐min block, we conducted a scan sample of the entire harem and recorded the behavior and nearest neighbor (i.e., an individual within one body length) of each member of the harem. We obtained 290.5 contact hours for the pre‐drought period in 2021, (hereafter “pre‐drought”) and 221.5 contact hours for 2022 (hereafter “late drought”) (Table S3).

All video was coded for behaviors of interest using ELAN video annotation software (ELAN (Version 6.7) [Computer Software], 2023). To investigate changes in activity budget across years, we coded 1–3 equally spaced 5‐min subsamples from each video (i.e., 5–10, 15–20, and 25–30 min), depending on the video length. The nearest neighbor of the focal animal was scored every 5 min. Within the subsamples, we counted steps per minute. We also scored all activities performed by the focal animal during the subsamples to construct time budgets (Table 2). Social behaviors were grouped into two broad categories, “passive socializing”, which were defined as proximity‐based social behaviors produced concurrently with a self‐maintenance behavior (i.e., resting in physical contact, grazing side‐by‐side), and “active socializing”, which included dedicated social behaviors with no additional self‐maintenance function (e.g., aggression, affiliation, etc.).

**TABLE 2:** Behaviors coded for the activity budget.

Behavior	Operational definition
Self‐maintenance behaviors	
Drinking	Consuming water
Grazing	Lowering head to consume grass, either stationary or while slowly perambulating
Geophagy	Lowering head to consume dirt/sand and then standing while masticating the material
Elimination	Urinating or defecating while not performing any other activity (i.e., travel or grazing)
Olfactory inspection	Either (a) lowering head to sniff urination or defecation, or (b) performing flehmen, in which upper lip is lifted and everted
Traveling	Lifting the head and taking > 5 steps
Vigilant	Standing with the head held above the 45° angle, all four legs planted on the ground, ears pricked forward, and head oriented toward the object of interest
Resting	Either (a) standing with the head at or below the 45° angle *and* eyes closed or half closed, ears backward or drooping to the side, one hind leg cocked and possibly lower lip drooping; or (b) laying in sternal or lateral recumbency
Hygiene	Auto‐grooming, rolling in dust, rubbing against an object, biting or kicking at flies, etc.
Standing	Standing with the head at a 45° angle but showing no additional signs of resting
Other	Behaviors which were ambiguous or seen infrequently enough that they did not warrant a full category. Nursing was also added to this category
Passive social behaviors: proximity‐based social behaviors or those performed while engaging in another self‐maintenance behavior	
Social grazing	Grazing within one body length of another member of the harem, most often in parallel
Social rest	Resting within one body length of another member of the harem, with or without physical contact (e.g., head resting on social partner). This is most often with both individuals standing, either parallel or in reverse parallel configuration, though in some cases one party will lay on the ground while the other stands beside or with neck directly over their social partner. Rarely, both will lay down side by side
Active social behaviors: social interactions that are not conducted concurrent with other self‐maintenance behaviors	
Affiliation	Engaging in non‐aggressive physical contact, including allogrooming, licking, and head rubbing
Aggression	Aggressive physical contact (e.g., displacement, rump swing/backing toward partner with flagging tail, biting, kicking, or threats to engage in these behaviors) produced with ears flattened against neck
Greeting	Produced at the meeting between two individuals of different social groups, for example, between harems, harem and bachelor group, etc. Nose‐to‐nose contact between individuals, which may or may not be accompanied by jaw snapping behavior. Greeting can progress to include other non‐aggressive behaviors, such as nose‐to‐genital inspection, nose‐to‐genital thrusting, etc.
Harem maintenance	Stallion lowering his head into the stereotyped “herding posture” (i.e., ears back, tail still, and neck extended down nearly touching the ground) and attempting to direct the females of his harem away from perceived threats. Could escalate into physical aggression with a bite
Sex	Courtship behaviors (e.g., male resting head on female's rump with chest against haunches, mutual anogenital region inspection, nose‐to‐nose snapping, mutual rubbing etc.) and copulation
Play	Physical interactions (e.g., chasing, biting, holding partner with mouth, blocking, rearing, spinning, neck wrestling, etc.) which are *not* produced concurrently with aggressive signals, namely, flat ears, flagging tail, and kicking, which are often thought to terminate a play fight rather than being part of play (McDonnell & Poulin 2002)

To analyze multimodal communication, we coded all interactions captured by our focal follow videos. Our coding scheme is published in detail elsewhere (Hex and Rubenstein 2024). In brief, we coded all concurrent visual, acoustic, tactile, and/or chemical signals produced (ethogram in Table S4). We only coded interactions in which the video was zoomed in enough for reliable coding of all areas of interest, and in which the focal individual was in focus and entirely in frame, unobscured by foliage or non‐focal animals. We eliminated 263 interactions from pre‐drought and 118 interactions from late drought which did not meet this criterion. To improve the interpretability of our results, we cleaned our data prior to analysis by removing multimodal postures if they contained a signaling component observed in fewer than two interactions. After cleaning, we were left with 336 high quality interactions comprising 1461 multimodal postures for pre‐drought and 115 interactions comprising 439 multimodal postures for late drought.

### 2.4 Data Analysis

Statistical analyses were conducted using R (v. 4.3.1). Significance for all tests was set to *α* = 0.05, and the ΔAIC cut‐off was < 2 for all models. AIC_c_ values corrected for small sample size were compared to determine which model best explained the variance using the “AICcmodavg” R package (Mazerolle 2020). We compared the top performing model to the null model using the “anova” function in “car” (Fox and Weisberg 2019). For all models, individual and harem ID were included as nested random effects. All network analyses were conducted using the igraph package (Fox and Weisberg 2019), and networks were visualized using the ggnetwork (Csardi and Nepusz 2006; Briatte, 2016).

#### 2.4.1 Body Condition and Survival

To quantify the effect of the drought on individuals, we compared body condition across years using a repeated measures Mann–Whitney *U* test. If the behavioral responses of individuals were insufficient to buffer them from the effects of drought, we predict a decrease in body condition in response to the drought.

#### 2.4.2 Activity Budget by Age/Sex Class

To evaluate how individuals modified their activities, we calculated an activity budget for each focal individual and used this individual data to produce the overall activity budget of the population for each year. We compared activity during the pre‐ and late drought periods by conducting a separate Mann–Whitney *U* test for each behavior. To probe how age/sex class might have influenced the changes observed across years, we ran a series of generalized linear mixed models (GLMMs) with a beta distribution (Salinas Ruíz et al. 2023) using the “glmmTMB” package (Brooks et al. 2017). The percentage of time engaged in the behavior was set as the response variable, while the interaction between age/sex class and year was set as the fixed effect. Each behavior was analyzed with a separate model, with the exception of “play” and “harem maintenance”, which were performed exclusively by juveniles and stallions, respectively. Sample size was insufficient to run GLMMs for “geophagy”, “other”, and “drinking”, and so were only analyzed with a Mann–Whitney *U*.

To further quantify how foraging effort changed from the pre‐ to the late drought periods, we constructed a linear mixed effect model (LMM), in which year and age/sex class were designated as the fixed effects and steps/min was set as the response variable as a proxy for the amount of movement individuals made in search of patches of food. We conducted a square‐root transformation to normalize the distribution and meet the assumptions of homogeneity of variance and normality of residuals.

#### 2.4.3 Intra‐Harem Social Networks

To evaluate how individuals changed their patterns of intra‐harem social associations to manage feeding competition during the drought, we constructed two nearest neighbor social networks for each harem, one from each time period. Due to high infant mortality in individuals under 1 year of age, we only included individuals older than 1 year in harem social networks. For each individual, we computed four measures of social connectivity for the pre‐ and late drought periods. First, we calculated degree, the number of social connections an individual had, to assess whether individuals showed changes in their social connectivity. We then calculated an index of association for each dyad within a harem during the pre‐ and late drought period, computed as a simple ratio index (SRI), using the formula:

$$\frac{x}{y^{a}+y^{b}+y^{\mathrm{ab}}+x}$$

wherein *x* is the number of sampling periods in which “*a*” and “*b*” were seen in association, *y*_*a*_ is the number of sampling periods in which “*a*” was observed alone, *y*_*b*_ is the number of sampling periods in which “*b*” was observed alone, and *y*_*ab*_ is the number of sampling periods in which “*a*” and “*b*” were both observed but not in association. For each individual, we then averaged all of their dyadic SRI scores to yield an average SRI, reflecting the average strength of their social bonds. Finally, the dyadic SRI values for each individual were used to calculate a coefficient of variance (CV), giving us a measure of variance of an individual's bond strengths.

We ran a series of Wilcoxon signed‐rank tests on change of these four measures (degree, dyadic SRI, average SRI, and CV for association strengths), eliminating individuals who were not present across both years. While Mantel's tests are frequently used to determine if bond strength is correlated across time (Farine and Whitehead 2015; Silva, Rangeewa, and Kryazhimskiy 2011), we determined that the meaningfulness of this analysis would be limited due to the relatively small average size of plains zebra harems (3–5 individuals). Therefore, we instead ran a simple linear regression to evaluate whether there was a relationship between the strength of bond between a dyad across years.

To capture higher level changes of intra‐harem connectivity, we also computed two harem‐level network measures. We first computed network density, the number of possible connections between individuals that were observed. Density ranges from 0 (a perfectly unconnected network) to 1 (a perfectly connected network). Density was compared across years using a Wilcoxon signed rank test. Second, using the dyadic SRI scores, we calculated the CV of the association strengths for each harem to determine if there was evidence for preferred associations between individuals within a harem. To do this, we first generated 1000 randomized networks for each harem, randomizing edges and their associated SRI but the holding number of edges and nodes constant, and then computed the CV for the resulting randomized networks to obtain a distribution of CVs. Significance was determined by computing how many of the resulting randomized CVs were higher than the observed value, divided by the number of randomizations (Farine and Whitehead 2015). If individuals have preferred associates, we would expect the observed CV to be significantly higher than the randomizations. However, this measure likely has limited meaningfulness in smaller harems (≤ 4 individuals), in which it could be possible to form relatively strong bonds with all members. Therefore, we limited this analysis to harems with > 4 individuals (*N* = 11 in 2021, *N* = 9 in late drought). Using harem‐level CV, we could determine whether harems became more heterogenous in bond strength in response to the drought by using a Wilcoxon signed rank test.

#### 2.4.4 Rate of Socializing

To evaluate whether there was an overall decrease in the rate of active socializing, we ran a Mann–Whitney U test on the number of social interactions/30 min, in which social interactions were defined as the number of *active* social interactions. We fitted an LMM to investigate whether the rate of active socializing was explainable by the intensity of drought and/or age/sex class. Year and age/sex class were designated as the fixed effects, and we examined every combination, including the interaction and a model with only random effects. We compared the top performing model to the null model using the “anova” function in “car” (Mazerolle 2020). We conducted an additional, post hoc chi‐square analysis to determine if juveniles shifted from primarily interacting with their mothers to other members of the harem during the drought.

#### 2.4.5 Multimodal Signaling Behavior

To assess how multimodal communication changed in response to the drought alongside other modifications to social behavior, we leveraged a network‐based method (Hex and Rubenstein 2024). Using networks to analyze multimodal communication can provide novel insights into the structure of social interactions. By taking advantage of the robust descriptive measures developed within network theory and uniting them with a theoretical framework adapting them for networks composed of signals, this technique can be used to test hypotheses about how communication responds to changes in the social environment.

Multimodal communication networks were constructed for each year following the methods outlined elsewhere (Hex and Rubenstein 2024). We generated subgraphs which excluded the highly central “neutral state” postures (i.e., “eyes open,” “upper lip normal,” “lower jaw closed,” “corner of lip normal,” “neck normal,” and “tail switching”), to improve visibility of the connections between other active components.

Component diversity was determined by computing the number of connected nodes in the network. We descriptively evaluated whether any signals were not seen in the late drought. To determine if the observed diversity in late drought was statistically different than would be expected due to random subsampling, we implemented a jackknifing procedure in which we took random subsamples from the pre‐drought dataset equal in size to that of the late drought period (*N* = 439) and computed the component diversity of the resulting subset (Farine and Whitehead 2015). We ran 1000 permutations of this resampling procedure and determined significance by calculating what proportion of the randomized diversity values were greater than or less than our observed diversity for late drought. Significance was two‐tailed and was set at the 0.975 and 0.025 percentiles for significantly greater or less than chance, respectively (Araya‐Salas et al. 2017; Kern and Radford 2018).

To evaluate the combinatorial flexibility of their multimodal communication, we computed the network density, correcting to eliminate mutually exclusive co‐occurrences within parts of the body (e.g., there could never be a connection between “ears forward” and “ears backwards”) (Hex and Rubenstein 2024). To do this, we first used the built‐in density function in igraph to represent all *theoretically possible* connections. Then we manually calculated the number of potential connections within each morphological component (e.g., ears forward, backwards, sideways, etc.) using the following formula:

$$\text{Potential conections}=\frac{n\times \left(n-1\right)}{2}$$

wherein *n* is the number of potential states a given mutually exclusive morphological component, such as ear or eye position, can be. We summed the results of all components to generate the number of *impossible* connections within the network. Finally, this was subtracted from the number of theoretically possible connections to produce the observed adjusted density.

We conducted a randomized subsampling procedure to determine if network density in late drought was significantly different than what would be expected simply due to a reduction in the number of nodes. To do so, we took 1000 subsamples of the complete pre‐drought multimodal network comprising randomly selected nodes equal to the number observed in the late drought and recalculated the adjusted density for each iteration. This allowed us to randomize the nodes in the subsample while preserving their connectivity, reflecting a null model in which there was only a reduction in repertoire size without a decrease in the combinatorial flexibility of the signals that remained. Significance was determined as described above.

We used the fast‐greedy clustering algorithm to classify components into modules, in which components within a module co‐occur at a higher frequency than those across modules (Hex and Rubenstein 2024). Using a clustering algorithm in this way can enable us to infer the communicative context in which a given component tends to be deployed based purely on patterns of co‐occurrence. Previously, it has been demonstrated that this method yields biologically meaningful modules composed of components that naturally occur in coherent communicative contexts (Hex and Rubenstein 2024). We then descriptively compared module membership across years to evaluate how patterns of combinatorial co‐occurrence in multimodal communication might have shifted to reflect changing communicative priorities.

We computed the modularity score of each year to assess whether communication became less combinatorically flexible and more stereotyped as the drought intensified. The modularity score, which ranges from 0 to 1, is a measure of the interconnectivity of a network (Clauset, Newman, and Moore 2004). When applied to a communication network, the modularity score can be used as another way to characterize the flexibility of a signaling system. A high modularity score (> 0.3) would indicate a repertoire comprising relatively discrete modules, in which there is high connectivity between signals within modules and little combinatorial co‐occurrences between signals across modules. In theory, this would reflect a relatively inflexible repertoire characterized by higher stereotypy and reduced ambiguity, as any given signal occurs in a smaller set of possible combinations. By contrast, a low modularity score (< 0.3), like that seen in plains zebras (Hex and Rubenstein 2024), would reflect a graded, combinatorically and contextually flexible repertoire in which components can appear in a variety of combinations and contexts both within and across modules. However, with this increased combinatorial flexibility could also come the potential for ambiguity and potential misunderstandings, as signals could co‐occur in a larger array of possible combinations.

## 3 Results

### 3.1 Zebras Showed no Change in Body Condition During the Drought

**FIGURE 2:**
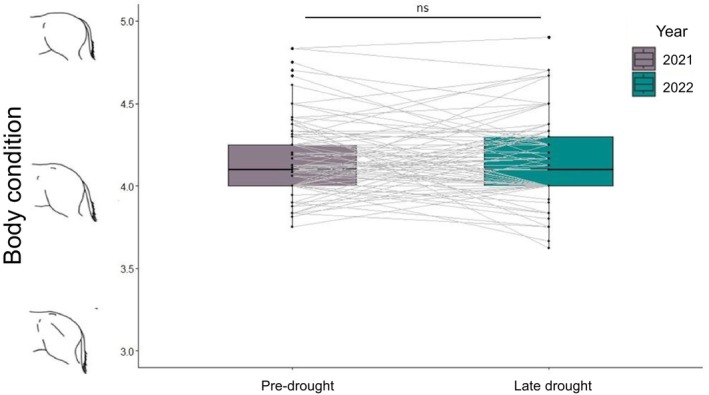
Individuals showed no change in body condition across the pre‐ (2021) and late (2022) drought periods.

In spite of the drought, individuals showed no difference in body condition (pre‐drought median: 4.1; late drought median: 4.1; Mann–Whitney *U*, *p* = 0.639, *z* = 0.05) (Figure 2). Between pre‐drought and late drought, 3 of our 109 adult focal individuals died. All of these individuals were females, aged 11, 12, and 22.

### 3.2 Zebras Increased Time Spent Foraging to the Detriment of Resting and Vigilance

**FIGURE 3:**
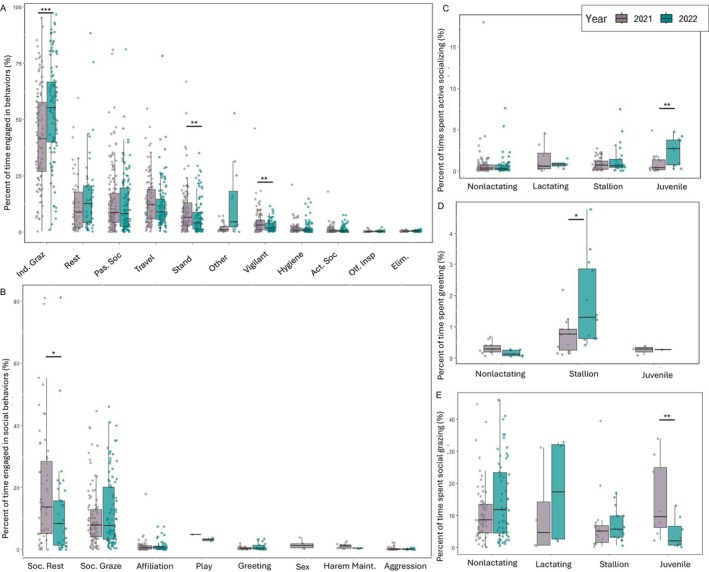
Percent of time devoted to activities in the pre‐ (2021) and late (2022) drought periods. (A) General activity budget observed across years, with social behaviors combined into “active socializing” and “passive socializing.” Act. Soc, “active socializing”; Elim., “Elimination”; Ind. Graz, “independent grazing”; Olf. Insp, “olfactory inspection”; Pass. Soc, “passive socializing”. (B) Percent of activity budget dedicated to both active and passive social behaviors. Harem maintenance, “harem maintenance”; Soc. Graze, “social grazing”; Soc. Rest, “social rest”. (C) “Active socializing”, in which juveniles showed a significant increase during the drought, (D) “Greeting”, in which stallions showed a significant increase during the drought, and (E) “Social grazing”, in which juveniles showed a significant decrease during the drought. * < 0.05, ** < 0.01, *** < 0.001.

We found that individuals engaged in significantly more grazing without social partners in close proximity (“independent grazing”) during the late drought (pre‐drought: 42% ± 21.8%; late drought: 54% ± 22.22%, Mann–Whitney *U*: *p* < 0.001, *z* = 0.262). In contrast, individuals spent significantly less time standing (pre‐drought: 9.8% ± 10.62%; late drought: 7.1% ± 7.76%, Mann–Whitney *U*: *p* = 0.0089, *z* = 0.183) and being vigilant (pre‐drought: 4.6% ± 5.77%; late drought: 2.6% ± 2.46%, Mann–Whitney *U*: *p* = 0.0082, *z* = 0.196) (Figure 3; Table S5). There were no significant differences in any other self‐maintenance behaviors across years, nor were there any significant interactions between year and age/sex class (GLMM outputs S1).

The best model to explain differences in steps/min included only year as a fixed effect and explained 99% of the variance (LMM outputs S2). Individuals took significantly more steps per minute in the late drought period (LMM: Estimate = 3.466e‐01, SE = 5.391e‐01, *t* = 6.43, *p* < 0.001*;* Figure S1).

### 3.3 Time Devoted to Socializing Did Not Decrease During Drought and Increased for Some Age/Sex Classes

Time spent resting in physical proximity to another individual (“social resting”) significantly decreased in late drought (pre‐drought: 19.01% ± 18.97%; late drought: 12.53% ± 16.49%, Mann–Whitney *U*: *p* = 0.0536, *z* = 0.204) (Figure 2). However, there was no significant difference in the amount of time individuals spent grazing in physical proximity of a social partner (“social grazing”) between years, though there was a significant interaction between age/sex class and year for juveniles, who spent significantly less time social grazing in the late drought period (GLMM: Estimate = −1.396, SE = 0.447, *z* = −3.124, *p* = 0.00178) (Figure 2).

The overall amount of active socializing did not significantly differ between years, and there was a significant interaction between year and age/sex class for juveniles and stallions. Juveniles engaged in significantly more general active socializing in late drought (GLMM: Estimate = 1.049, SE = 0.359, *z* = 2.92, *p* = 0.00346). Stallions engaged in significantly more greeting behaviors with extra‐harem members, particularly males, in the late drought (GLMM: Estimate = 1.164, SE = 0.471, *z* = 2.470, *p* = 0.0135) (Figure 2).

### 3.4 Zebras Did Not Reduce Strength or Number of Social Bonds and Harems Became More Homogenous in Bond Strength

We found no significant difference in density of harem proximity networks across years (Wilcoxon signed rank test: *p* = 0.636, *z* = 0.126). However, harem‐level CV for bond strength significantly decreased in the late drought (Wilcoxon signed rank test: *p* = 0.0244, *z* = 0.607) (Figure S2; Table S6). There was no significant difference in any individual measure of social connectivity across years (Wilcoxon signed rank test: dyadic SR *p* = 0.785, *z* = 0.0139; average individual SRI *p* = 0.333, effect size *z* = 0.0977; individual CV of bond strength *p* = 0.316, *z* = 0.112; degree *p* = 0.168, *z* = 0.118) (Figure S3). There was a significant positive relationship between dyadic SRI across years, and the strength of the bond in pre‐drought was predictive of the strength of the bond in late drought (linear regression: *p* < 0.001, *R* ^2^ = 0.73 ± 0.0193) (Figure S4).

### 3.5 Zebras Engaged in Less Frequent Active Social Interactions

**FIGURE 4:**
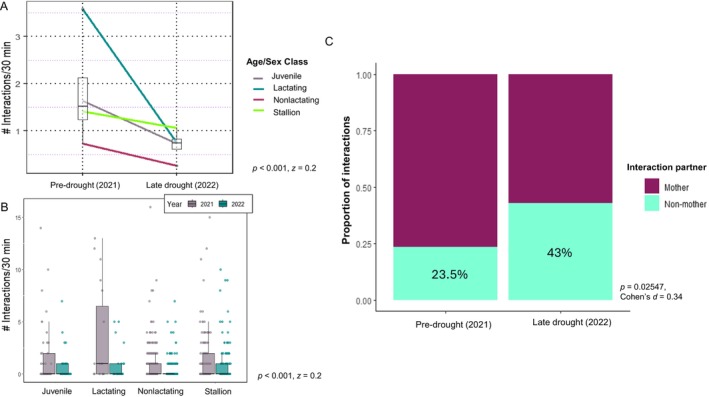
(A) Mean change in the number of social interactions across years by age/sex class. Box plot indicates the combined median number of interactions per 30 min. (B) Number of active social interactions by age/sex class. Females had the highest decrease in the active socializing rate (*p <* 0.001). (C) Proportion of interactions juveniles had with mothers and non‐maternal social partners within the harem in the pre‐drought (2021) and late drought (2022) periods.

In the pre‐drought, 40.3% of 30‐min observation periods had at least one active social interaction, decreasing to only 21.7% of observation periods in the late drought (pre‐drought: 1.05 ± 2.13 interactions/30 min; 2022: 0.526 ± 1.37 interactions/30 min, Mann–Whitney *U*: *p* < 0.001, *z* = 0.2). Our top performing model revealed a significant interaction between year and age/sex class (LMM output S3). Lactating females had a significantly lower rate of active social interaction in the late drought period than in the pre‐drought period (LMM: Estimate = −1.35235, SE = 0.30852, *t* = −4.383, *p* < 0.001) (Figure 4, Table S7).

As juveniles spent more time engaged in active socializing in the late drought, we conducted an additional, post hoc chi‐square analysis to determine if juveniles shifted from primarily interacting with their mothers to other members of the harem during the drought. We found that juveniles interacted with non‐maternal social partners significantly more often in the late drought period (*X* ^2^(*df* = 1) = 4.99, *p* = 0.02547, Cohen's *d* = 0.34), from 23.5% in pre‐drought to 43% of interactions in late drought (Figure 4).

### 3.6 Repertoires Became Less Diverse and More Simplified During Drought Especially in the Contexts of Aggression and Greeting

**FIGURE 5:**
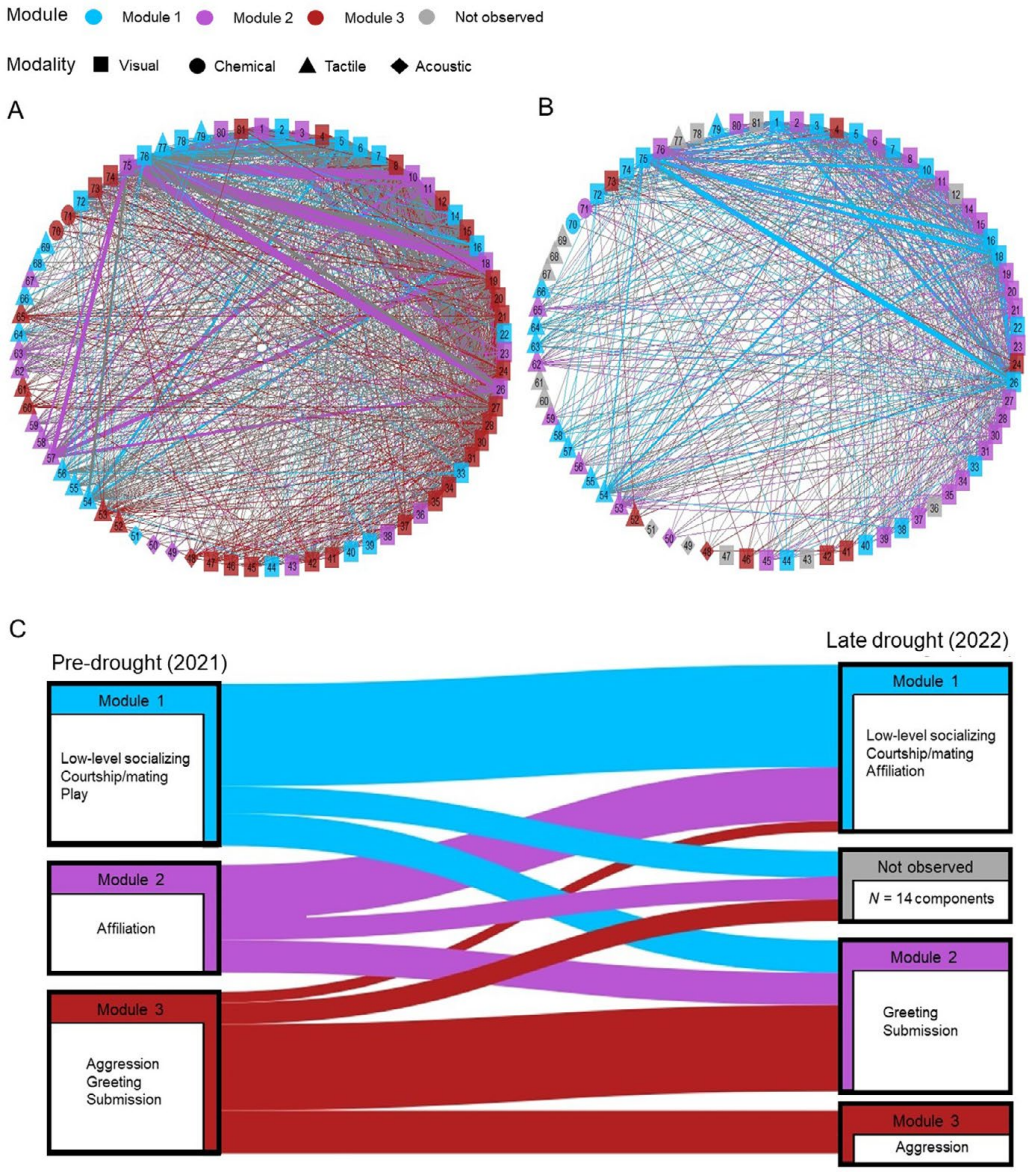
Connectivity of multimodal communication networks changed in connectivity and density across years. (A) Pre‐drought (2021) and (B) late drought (2022). Thickness of edges between signals, represented as nodes, reflects their frequency of co‐occurrence. The color of a node indicates its module, in which all signals co‐occur at a higher frequency than they do with those in other modules. Colored edges indicate within‐module co‐occurrences, and gray edges indicate between‐module co‐occurrences. Node labels can be found in Table S6. (C) Change in module membership across years from the pre‐ to late drought, including signal components not observed in late drought. The contexts grouped within each module are listed.

The diversity of signals observed decreased from 81 signal components in pre‐drought to 67 signal components in late drought (*p* < 0.001, randomized diversity mean = 74.94 ± 1.8, 95% CI [74.83–75.05], randomized range = 69–80) (Figure 5). Lost signals represented 50% of vocal signals (*N* = 2/4), 35% of tactile signals (*N* = 7/20), and 9% of visual signals (*N* = 5/55) (Table S8).

The density, or proportion of possible combinatorial co‐occurrences that were actually observed in the multimodal communication network, significantly decreased from 0.48 in the pre‐drought period to 0.44 in the late drought period (*p* = 0.012, randomized density mean = 0.505 ± 0.3, 95% CI [0.503–0.507], randomized range = 0.408–0.589), reflecting that individuals not only used a smaller repertoire, but also used this smaller repertoire with less combinatorial flexibility.

Modularity score, a measure of network connectivity and signal context specificity, was relatively low across both years, in line with previous studies (Hex and Rubenstein 2024). Modularity decreased slightly in late drought, from 0.056 in the pre‐drought to 0.043 in the late drought. Though the number of modules did not change across years, module membership shifted, reflecting a change in patterns of co‐occurrence between components. Most notably, the module which comprised the spectrum of behaviors ranging from aggression to greeting and submission was split in two in the late drought network (Figure 5). Meanwhile, behaviors seen in affiliative interactions, which comprised their own module in pre‐drought, joined the module composed of components observed in low‐level socializing, courtship, and mating in the late drought network. Of the 14 signals not observed in late drought, five were a part of the aggression module in pre‐drought, four were in the affiliation module, and five were seen in the low‐level association module.

## 4 Discussion

As human activities continue to vault planetary temperatures, curb rainfall, and provoke drought, organisms from the lowest trophic levels to the highest will face increased pressures to their survival. Understanding how animals respond to these pressures is critical for conservation and management. Here, we explored the effect of severe drought on plains zebra associations and interactions with conspecifics, and how communication shifted in response to harsh environmental conditions.

In spite of the drought, we saw no change in individual body condition, and only three adult individuals died. This is in contrast to reports of high mortality during the drought in other similarly sized ungulates on the landscape (Miwu et al. 2022). This may have been partially facilitated by plains zebras increasing the amount of time they devoted to independent grazing at the expense of time spent standing, being vigilant, and engaging in social rest. They also took more steps/min, reflecting increased foraging effort in search of palatable pastures. These findings suggest that individuals generally reapportioned time away from energy conserving behaviors toward foraging to maintain body condition during the drought.

There was a significant decrease in the frequency and number of active social interactions observed in the late drought period. This was particularly true for lactating mothers, who have the greatest physiological need (Fischhoff et al. 2007). However this overall reduction in interaction *frequenc*y was not reflected in significant change in the amount of *time* individuals spent actively socializing. Indeed, in certain age/sex classes, individuals increased the time devoted to certain types of active social behaviors. Stallions devoted significantly more time to greeting behavior during the late drought period, but with no concurrent increase in aggressive or harem tending behaviors. Stallions are the primary social shield between their harem and the outside social world, particularly bachelor male harassment (Rubenstein and Nuñez 2009; Rubenstein 2010). Mares prefer stallions who will afford them more time to graze by directly engaging with other males rather than driving the harem away from potential social threats (Rubenstein and Nuñez 2009), and previous work has shown females can gain up to 2 h of grazing a day when they have a dominant stallion to provide vigilance and protection (Rubenstein 1993). The current study is unable to determine whether stallions were responding to an increased perceived threat in the social landscape, or to some other proximate cue within the harem during the drought. However, the lack of increase in rates of aggression, harem tending, or sexual behaviors during the drought make it unlikely that stallions were responding to increased bachelor pressure. Nonetheless, by engaging in more greeting behaviors with bachelors and other stallions, stallions may have been able to reinforce dominance relationships and maintain familiarity with tolerated, allied males, and in doing so simultaneously reduce the risk of cuckoldry and providing females time to graze (Rubenstein and Hack 2004). These findings suggest that stallions and the social role they fill may provide a critical service to the rest of the harem, especially during times of acute environmental hardship.

Juveniles spent significantly less time social grazing and more time engaged in active socializing during the late drought, likely by engaging in longer, less frequent bouts of interaction. This finding cannot be explained by maturational effects, as only one foal was present in our sample across both study periods, and there was no difference in the average age of focal juveniles during the pre‐ and late drought periods. Paired with the decrease in both number and amount of time devoted to interactions by lactating females, these findings suggest an intriguing conflict of interest between mothers and their offspring. In the pre‐drought period, lactating females had the highest interaction frequency of any age/sex class, engaging in 3.58 ± 4.66 interactions/30 min. Most of these interactions occurred during nursing and comprised tactile exchanges with their young, such as licking, rubbing, and allogrooming. However, in the late drought period, we witnessed fewer active interactions between mothers and their foals as mares focused their attention on grazing, possibly prioritizing their own survival and potential future reproductive opportunities over socializing their foals (Hrdy 1999; Clutton‐Brock 1991).

Juveniles appeared to nonetheless compensate for the lack of maternal input by engaging other members of the harem, particularly nonlactating females. Alloparental care has been found to buffer mothers by allowing them to reduce their own investment without compromising offspring fitness (Fite et al. 2005; Hammers et al. 2021; Guindre‐Parker and Rubenstein 2018) and harsher climates can promote greater cooperative childcare (Martin et al. 2020; Raviv et al. 2023). Though not traditionally considered to be a cooperatively breeding species, non‐maternal members of plains zebra harems provide indirect alloparental care to juveniles in the form of vigilance, protection, and play (Klingel 1965; Hex et al. 2022) (Movie S1). Future investigation could elucidate whether this shift in social partners was driven by juveniles seeking social interaction in the absence of typical maternal input, or whether non‐maternal social partners initiated contact.

Plains zebra intra‐harem nearest neighbor networks were remarkably resilient, mirroring previous studies which have found no apparent effect of drought on social bonds (Waterman and Fenton 2000; Godfrey, Sih, and Bull 2013). Individuals showed no significant change in their strength or number of social bonds, and dyadic bond strength remained consistent across years, thus providing no support for hypotheses H_1_ or H_2_ (Table 1). However, harems became significantly more homogenous in bond strength during the drought, with fewer harems showing evidence for statistically preferred associates in late drought. This evidence suggests that individuals may slightly divest in the amount of time spent near their closest associates while maintaining weaker bonds, in support of H_3_. However, the majority of tested network measures remained unchanged, most strongly supporting the hypothesis that feeding competition was not severe enough to impose a large cost on social proximity (H_4_). These findings are recapitulated by the lack of significant difference in the amount of time spent in social grazing without a loss of body condition across years. It is possible that our findings could have also been influenced by different individuals adopting alternative strategies in accordance with their priorities. For instance, the greater nutritive needs of lactating females may have induced them to spend more time apart from other harem members during the drought than nonlactating females. Future research could further elucidate such state‐dependent strategies. Nonetheless, the ability to engage in passive social behaviors, such as grazing in close physical proximity, without apparent feeding competition may allow plains zebras to both meet their increased nutritive demands and reduce their frequency of active social interaction without having to compromise intra‐harem connectivity.

Finally, the structure of multimodal communication shifted significantly in response to the drought. Individuals not only used a less diverse repertoire, particularly tactile signals and those observed in high levels of aggression (e.g., “bite ankles”) or play (e.g., “neck wrestle”) (Table S6), but also deployed a smaller variety of multimodal combinations in their signaling behavior. Module membership also shifted substantially, with the aggression module becoming smaller and less diverse in late drought. This redistribution of module membership reveals a change in patterns and frequency of co‐occurrence between components, particularly in the relatively energetically costly and high‐risk contexts of aggression, greeting, and submission. In pre‐drought, aggressive signals occurred in a single module with those used in greetings and submission. This clustering reflects naturalistic interactions in which greetings exists on a spectrum depending upon the relative familiarity and dominance of participants (Hex and Rubenstein 2024). Particularly in males, greeting interactions can be protracted and complex, involving many parties, and will occasionally escalate into higher level disputes when dominance cannot be established (Movie S2).

The splitting of the aggression module into two, one with aggressive signals, and another with greeting and submission, suggests a fundamental change in how greeting rituals were navigated under the regime of drought. The dramatically reduced aggression module contained only the most context‐specific, aggressive signals (Hex and Rubenstein 2024), suggesting that when individuals engaged in aggression, they did so with a simplified repertoire that could convey their message with minimal potential for misunderstanding. Meanwhile, the clustering of greeting and submissive components into a new module suggests that these encounters were less likely to grade into aggression during the drought. Indeed, we observed no increase in aggressive interactions, even as the number of greeting interactions significantly increased. Therefore, even though stallions increased their greeting activities, they may have been able to modulate their energy expenditure by altering how they engaged in these interactions to avoid conflict. Taken together with our finding that repertoire size and combinatorial flexibility decreased, these changes in repertoire use would make it easier for individuals to efficiently predict their partner's motivation and react appropriately. When time is the only factor limiting an individual's ability to access resources and social interactions cannot be eliminated, reducing the complexity of interactions could minimize their risk by enabling conflicts to more effectively avoided.

Overall, these data reveal how individuals respond to acute environmental stress by flexibly modifying their activity budgets and communication without compromising the strength of their social bonds. Further, our findings suggest that the differentiated social roles within the harem may have enabled all individuals, including those with the most need, to reach their subsistence requirements and maintain body condition. We here acknowledge the important caveat that the present study represents the comparison of two time points collected during the same time of the year, one in a more typical dry season, and one during an unusually severe drought. However, we do not yet know whether and the extent to which the behaviors we investigated shift across years in the absence of an environmental perturbation like drought, nor the effect of intra‐annual cycles of seasonal variation. Future investigation comparing activity budges, intra‐harem social networks, and multimodal communication at different times of the year, as well as the continuation of longitudinal data collection across more years, will enable us to further contextualize our findings within the scope of expected behavioral variation.

Our findings have implications for the “Social Buffering Hypothesis”, which proposes that social bonds can enable individuals to endure adversity by insulating them from the deleterious effects of social and/or environmental stress (Beery and Kaufer 2015). Plains zebras suffered comparatively less mortality during this severe drought (~0.01% of the population of Laikipia county) than the sympatric Grévy's zebra (2% of the population), despite the latter being more xeric‐adapted (Kinnaird and O'Brien 2012; Gersick and Rubenstein 2017). However, unlike plains zebras, Grévy's zebras live in fission–fusion societies, in which females form ephemeral bonds as they wander between the territories of solitary males, and thus may not be able to benefit from cooperation under harsh conditions (Klingel 1974; Gersick and Rubenstein 2017). Cooperation and group living has been found to enable species to inhabit and persist in harsh environments (Rubenstein and Lovette 2007; Jetz and Rubenstein 2011; Cornwallis et al. 2017; Firman et al. 2020; Martin et al. 2020; Camerlenghi et al. 2024), and it has been proposed that sociality may enable animals to respond to a rapidly changing world (Komdeur and Ma 2021). It is possible that in plains zebras' stable, a cooperative social system contributes to their heightened resilience to particularly severe droughts compared to Grévy's zebras and other similarly sized ungulates on the landscape (Georgiadis, Hack, and Turpin 2003; Grange et al. 2004). While the current study lacks the requisite fitness data to truly demonstrate social buffering, the heterogenous, role‐specific behavioral changes we observed within the harem warrant further investigation to elucidate the influence of sociality on the resilience of plains zebras.

As climate change and its associated increases in extreme weather events are expected to continue in the Anthropocene, species whose physiology and social structure grants them the flexibility to behaviorally respond to shifting conditions are likely to be the ones with the capacity to endure in a human modified world (Komdeur and Ma 2021; Menzel and Feldmeyer 2021). The findings of this study contribute not only to our understanding of how the behavioral flexibility facilitated by sociality can enable individuals to respond to environmental stressors, but also more generally about the benefits of sociality in fluctuating and unpredictable environments (Komdeur and Ma 2021). While the combination of a species' physiology, social organization, and life history will influence how they can respond to unpredictable environmental conditions (Menzel and Feldmeyer 2021; Blumstein, Hayes, and Pinter‐Wollman 2023), those living in stable, cooperative social groups may possibly benefit from greater resilience to these fluctuations (Komdeur and Ma 2021). For some lineages, the benefits of social living under the new regime of unpredictable resource availability and precipitation may even increase the benefits of cooperation and group living, leading to social organizations shifting toward sociality (Ostwald, da Silva, and Seltmann 2024).

However, the current rate of environmental change exceeds historic rates, which may limit the extent to which we can expect modern responses to climate change to mirror those proposed to have resulted in the evolution of cooperation (Rubenstein and Lovette 2007; Jetz and Rubenstein 2011). Therefore, it is critical to continue investigating how individuals balance their self‐maintenance requirements with the constraints of their social environment in species that occupy different regimes of conflict and cooperation, such as those which engage in contest rather than scramble competition for resources, and in which the benefits of sociality may be outweighed by the costs. Only by investigating a diversity of species occupying different niches in the changing landscape can we develop a holistic understanding of how, and which, species have the capacity to survive as Earth's climate becomes increasingly extreme and unpredictable.

## Author Contributions

**Severine B. S. W. Hex:** conceptualization (lead), data curation (lead), formal analysis (lead), investigation (lead), methodology (lead), validation (lead), visualization (lead), writing – original draft (lead), writing – review and editing (lead). **Erin S. Isbilen:** conceptualization (supporting), investigation (supporting), writing – review and editing (equal). **Daniel I. Rubenstein:** conceptualization (equal), funding acquisition (lead), supervision (lead), writing – review and editing (equal).

## Conflicts of Interest

The authors declare no conflicts of interest.

## Supporting information


Data S1.

## Data Availability

Data available from the Dryad Digital Repository https://doi.org/10.5061/dryad.x0k6djht3.

## References

[ece370632-bib-0001] Araya‐Salas, M. , K. Wojczulanis‐Jakubas , E. M. Phillips , D. J. Mennill , and T. F. Wright . 2017. “To Overlap or Not to Overlap: Context‐Dependent Coordinated Singing in Lekking Long‐Billed Hermits.” Animal Behaviour 124: 57–64.

[ece370632-bib-0002] Beery, A. K. , and D. Kaufer . 2015. “Stress, Social Behavior, and Resilience: Insights From Rodents.” Neurobiology of Stress 1: 116–127. 25562050 10.1016/j.ynstr.2014.10.004 PMC4281833

[ece370632-bib-0003] Blumstein, D. T. , L. D. Hayes , and N. Pinter‐Wollman . 2023. “Social Consequences of Rapid Environmental Change.” Trends in Ecology & Evolution 38, no. 4: 337–345. 36473809 10.1016/j.tree.2022.11.005

[ece370632-bib-0004] Briatte, F. 2016. “ggnetwork: Geometries to Plot Networks with ‘ggplot2’.” R Package Version 0.5, 1.

[ece370632-bib-0005] Brooks, M. E. , K. Kristensen , K. J. van Benthem , et al. 2017. “glmmTMB Balances Speed and Flexibility Among Packages for Zero‐Inflated Generalized Linear Mixed Modeling.” R Journal 9, no. 2: 378–400. 10.32614/RJ-2017-066.

[ece370632-bib-0006] Buchholz, R. , J. D. Banusiewicz , S. Burgess , S. Crocker‐Buta , L. Eveland , and L. Fuller . 2019. “Behavioural Research Priorities for the Study of Animal Response to Climate Change.” Animal Behaviour 150: 127–137.

[ece370632-bib-0007] Camerlenghi, E. , S. Nolazco , D. R. Farine , R. D. Magrath , and A. Peters . 2024. “Social Restructuring During Harsh Environmental Conditions Promotes Cooperative Behaviour in a Songbird.” Proceedings of the Royal Society B 291: 20232427. 38628131 10.1098/rspb.2023.2427 PMC11022012

[ece370632-bib-0008] Cameron, E. Z. , T. H. Setsaas , and W. L. Linklater . 2009. “Social Bonds Between Unrelated Females Increase Reproductive Success in Feral Horses.” Proceedings of the National Academy of Sciences of the United States of America 106: 13850–13853. 19667179 10.1073/pnas.0900639106 PMC2728983

[ece370632-bib-0009] Carter, G. C. , D. R. Farine , and G. S. Wilkinson . 2017. “Social Bet‐Hedging in Vampire Bats.” Biology Letters 13: 10–13. 10.1098/rsbl.2017.0112 PMC5454239 28539459

[ece370632-bib-0010] Clauset, A. , M. E. J. Newman , and C. Moore . 2004. “Finding Community Structure in Very Large Networks.” Physical Review E 70: 066111. 10.1103/PhysRevE.70.066111 15697438

[ece370632-bib-0011] Clauss, M. 2013. “Digestive Physiology and Feeding Behaviour of Equids – A Comparative Approach.” In *Horse Health Nutrition – European Equine Health Nutrition Congress*. Gent, Belgium, 25–33.

[ece370632-bib-0012] Clutton‐Brock, T. H. 1991. The Evolution of Parental Care. Vol. 64. Princeton, NJ: Princeton University Press.

[ece370632-bib-0013] Cohen, S. , and T. A. Willis . 1985. “Stress, Social Support, and the Buffering Hypothesis.” Psychological Bulletin 98: 310–357. 3901065

[ece370632-bib-0014] Conradt, L. , T. H. Clutton‐Brock , and F. E. Guinness . 2000. “Sex Differences in Weather Sensitivity Can Cause Habitat Segregation: Red Deer as an Example.” Animal Behaviour 59: 1049–1060. 10860532 10.1006/anbe.2000.1409

[ece370632-bib-0015] Cornwallis, C. K. , C. A. Botero , D. R. Rubenstein , P. A. Downing , S. A. West , and A. S. Griffin . 2017. “Cooperation Facilitates the Colonization of Harsh Environments.” Nature Ecology & Evolution 1: 1–10. 28812731 10.1038/s41559-016-0057

[ece370632-bib-0016] Csardi, G. , and T. Nepusz . 2006. “The Igraph Software Package for Complex Network Research.” InterJournal, Complex Systems 1695: 1–9.

[ece370632-bib-0017] Cunningham, P. L. , and T. Wronski . 2011. “Seasonal Changes in Group Size and Composition of Arabian Sand Gazelle *Gazella subgutturosa marica* Thomas, 1897 During a Period of Drought in Central Western Saudi Arabia.” Current Zoology 57: 36–42.

[ece370632-bib-0018] ELAN (Version 6.7) [Computer Software] . 2023. “Nijmegen: Max Planck Institute for Psycholinguistics, The Language Archive.” https://archive.mpi.nl/tla/elan.

[ece370632-bib-0019] Farine, D. R. , and H. Whitehead . 2015. “Constructing, Conducting and Interpreting Animal Social Network Analysis.” Journal of Animal Ecology 84: 1144–1163. 26172345 10.1111/1365-2656.12418 PMC4973823

[ece370632-bib-0020] Fattorini, N. , S. Lovari , S. Franceschi , et al. 2023. “Animal Conflicts Escalate in a Warmer World.” Science of the Total Environment 871: 161789. 36716887 10.1016/j.scitotenv.2023.161789

[ece370632-bib-0021] Firman, R. C. , D. R. Rubenstein , J. M. Moran , K. C. Rowe , and B. A. Buzatto . 2020. “Extreme and Variable Climatic Conditions Drive the Evolution of Sociality in Australian Rodents.” Current Biology 30: 691–697.e3. 32008900 10.1016/j.cub.2019.12.012

[ece370632-bib-0022] Fischhoff, I. R. , S. R. Sundaresan , J. Cordingley , H. M. Larkin , M. J. Sellier , and D. I. Rubenstein . 2007. “Social Relationships and Reproductive State Influence Leadership Roles in Movements of Plains Zebra, *Equus Burchellii* .” Animal Behaviour 73: 825–831.

[ece370632-bib-0023] Fite, J. E. , K. J. Patera , J. A. French , M. Rukstalis , E. C. Hopkins , and C. N. Ross . 2005. “Opportunistic Mothers: Female Marmosets ( *Callithrix kuhlii* ) Reduce Their Investment in Offspring When They Have to, and When They Can.” Journal of Human Evolution 49: 122–142. 15935439 10.1016/j.jhevol.2005.04.003 PMC2987622

[ece370632-bib-0024] Foley, C. , N. Pettorelli , and L. Foley . 2008. “Severe Drought and Calf Survival in Elephants.” Biology Letters 4: 541–544. 18682358 10.1098/rsbl.2008.0370 PMC2610102

[ece370632-bib-0025] Fox, J. , and S. Weisberg . 2019. An R Companion to Applied Regression. 3rd ed. Thousand Oaks CA: Sage. https://socialsciences.mcmaster.ca/jfox/Books/Companion/.

[ece370632-bib-0026] Georgiadis, N. , M. Hack , and K. Turpin . 2003. “The Influence of Rainfall on Zebra Population Dynamics: Implications for Management.” Journal of Applied Ecology 40: 125–136.

[ece370632-bib-0027] Gersick, A. S. , and D. I. Rubenstein . 2017. “Physiology Modulates Social Flexibility and Collective Behaviour in Equids and Other Large Ungulates.” Philosophical Transactions of the Royal Society B 372: 20160241. 10.1098/rstb.2016.0241 PMC5498301 28673917

[ece370632-bib-0028] Ginsberg, J. R. 1988. “Social Organization and Mating Strategies of An Arid‐Adapted Ungulate: The Grevy's Zebra.” Doctoral diss., Princeton University.

[ece370632-bib-0029] Godfrey, S. S. , A. Sih , and C. M. Bull . 2013. “The Response of a Sleepy Lizard Social Network to Altered Ecological Conditions.” Animal Behaviour 86: 763–772.

[ece370632-bib-0030] Grange, S. , P. Duncan , J. M. Gaillard , et al. 2004. “What Limits the Serengeti Zebra Population?” Oecologia 140: 523–532. 15293043 10.1007/s00442-004-1567-6

[ece370632-bib-0031] Guindre‐Parker, S. , and D. R. Rubenstein . 2018. “Multiple Benefits of Alloparental Care in a Fluctuating Environment.” Royal Society Open Science 5: 172406. 29515910 10.1098/rsos.172406 PMC5830800

[ece370632-bib-0034] Hammers, M. , S. A. Kingma , L. A. van Boheemen , et al. 2021. “Helpers Compensate for Age‐Related Declines in Parental Care and Offspring Survival in a Cooperatively Breeding Bird.” Evolution Letters 5: 143–153. 33868710 10.1002/evl3.213 PMC8045936

[ece370632-bib-0036] Hex, S. B. , M. Mwangi , R. Warungu , and D. I. Rubenstein . 2022. “An Observation of Attempted Infanticide and Female – Female Cooperation in Wild Plains Zebras ( *Equus quagga* ).” Behaviour 159: 1341–1364.

[ece370632-bib-0037] Hex, S. B. , and D. I. Rubenstein . 2024. “Using Networks to Visualize, Analyse and Interpret Multimodal Communication.” Animal Behaviour 207: 295–317.

[ece370632-bib-0038] Hex, S. B. , K. J. Tombak , and D. I. Rubenstein . 2021. “A New Classification of Mammalian Uni‐Male Multi‐Female Groups Based on the Fundamental Principles Governing Inter‐ and Intrasexual Relationships.” Behavioral Ecology and Sociobiology 75: 157.

[ece370632-bib-0039] Holekamp, K. E. , J. E. Smith , C. C. Strelioff , R. C. Van Horn , and H. E. Watts . 2012. “Society, Demography and Genetic Structure in the Spotted Hyena.” Molecular Ecology 21: 613–632. 21880088 10.1111/j.1365-294X.2011.05240.x

[ece370632-bib-0040] Hrdy, S. B. 1999. Mother Nature: A History of Mothers, Infants and Natural Selection. New York: Pantheon Books. 10.1006/anbe.1999.1378 10792945

[ece370632-bib-0041] IPCC . 2014. Climate Change 2014: Synthesis Report. Contribution of Working Groups I, II and III to the Fifth Assessment Report of the Intergovernmental Panel on Climate Change. Edited by Core Writing Team, R. K. Pachauri and L. A. Meyer, pp. 151. Geneva, Switzerland: IPCC.

[ece370632-bib-0042] IPCC . 2023. “Summary for Policymakers.” In Climate Change 2023: Synthesis Report. Contribution of Working Groups I, II and III to the Sixth Assessment Report of the Intergovernmental Panel on Climate Change, edited by Core Writing Team , H. Lee , and J. Romero , 1–34. Geneva, Switzerland: IPCC.

[ece370632-bib-0043] Isbell, L. , and T. Young . 2002. “Ecological Models of Female Social Relationships in Primates: Similarities, Disparities, and Some Directions for Future Clarity.” Behaviour 139: 177–202.

[ece370632-bib-0044] Ismail, K. , K. Kamal , M. Plath , and T. Wronski . 2011. “Effects of an Exceptional Drought on Daily Activity Patterns, Reproductive Behaviour, and Reproductive Success of Reintroduced Arabian Oryx ( *Oryx leucoryx* ).” Journal of Arid Environments 75, no. 2: 125–131.

[ece370632-bib-0045] Jetz, W. , and D. R. Rubenstein . 2011. “Environmental Uncertainty and the Global Biogeography of Cooperative Breeding in Birds.” Current Biology 21: 72–78. 21185192 10.1016/j.cub.2010.11.075

[ece370632-bib-0046] Johnston, G. R. , E. J. Lanham , and C. M. Bull . 2020. “United in Adversity: Aridity and Cold Influence Aggregation Behaviour in a Social Lizard, *Egernia stokesii* .” Austral Ecology 45: 418–425.

[ece370632-bib-0047] Kern, J. M. , and A. N. Radford . 2018. “Experimental Evidence for Delayed Contingent Cooperation Among Wild Dwarf Mongooses.” Proceedings of the National Academy of Sciences 115: 6255–6260. 10.1073/pnas.1801000115 PMC6004489 29844179

[ece370632-bib-0048] Kinnaird, M. F. , and T. G. O'Brien . 2012. “Effects of Private‐Land Use, Livestock Management, and Human Tolerance on Diversity, Distribution, and Abundance of Large African Mammals.” Conservation Biology 26, no. 6: 1026–1039. 23082891 10.1111/j.1523-1739.2012.01942.x

[ece370632-bib-0049] Klingel, H. 1965. “The Social Organisation and Population Ecology of the Plains Zebra.” Zoologica Africana 4: 149–163.

[ece370632-bib-0050] Klingel, H. 1974. “A Comparison of the Social Behaviour of the Equidae.” In Behaviour of Ungulates and Its Relation to Management, edited by V. Geist and F. Walther , 124–132. Morges, Switzerland: IUCN Publications.

[ece370632-bib-0051] Komdeur, J. , and L. Ma . 2021. “Keeping Up With Environmental Change: The Importance of Sociality.” Ethology 127: 790–807. 10.1111/eth.13200.

[ece370632-bib-0052] Malonza, P. 2022. “Press Statement on the Impacts of the Current Drought on Wildlife in Kenya.” Kenyan Ministry of Tourism, Wildlife, and Heritage.

[ece370632-bib-0053] Marchand, P. , M. Garel , G. Bourgoin , D. Dubray , D. Maillard , and A. Loison . 2015. “Sex‐Specific Adjustments in Habitat Selection Contribute to Buffer Mouflon Against Summer Conditions.” Behavioral Ecology 26: 472–482.

[ece370632-bib-0054] Marshall, H. H. , J. L. Sanderson , F. Mwanghuya , et al. 2016. “Variable Ecological Conditions Promote Male Helping by Changing Banded Mongoose Group Composition.” Behavioral Ecology 27: 978–987. 27418750 10.1093/beheco/arw006 PMC4943108

[ece370632-bib-0055] Martin, J. S. , E. J. Ringen , P. Duda , and A. V. Jaeggi . 2020. “Harsh Environments Promote Alloparental Care Across Human Societies.” Proceedings of the Royal Society B: Biological Sciences 287: 20200758. 10.1098/rspb.2020.0758 PMC7482265 32811302

[ece370632-bib-0056] Mazerolle, M. J. 2020. “AICcmodavg: Model Selection and Multimodel Inference Based on (Q)AIC(c).” R Package Version 2.3‐1. https://cran.r‐project.org/package=AICcmodavg.

[ece370632-bib-0096] McDonnell, S. M. , and A. Poulin . 2002. “Equid play ethogram.” Applied Animal Behaviour Science 78, no. 2‐4: 263–290.

[ece370632-bib-0057] McFarland, R. , D. Murphy , D. Lusseau , et al. 2017. “The ‘Strength of Weak Ties’ Among Female Baboons: Fitness‐Related Benefits of Social Bonds.” Animal Behaviour 126: 101–106.

[ece370632-bib-0058] McMillan, N. A. , S. D. Fuhlendorf , B. Luttbeg , et al. 2022. “Bison Movements Change With Weather: Implications for Their Continued Conservation in the Anthropocene.” Ecology and Evolution 12: e9586. 36514548 10.1002/ece3.9586 PMC9731910

[ece370632-bib-0059] Menzel, F. , and B. Feldmeyer . 2021. “How Does Climate Change Affect Social Insects?” Current Opinion in Insect Science 46: 10–15. 33545433 10.1016/j.cois.2021.01.005

[ece370632-bib-0060] Miwu, S. , S. Ngene , P. Omondi , et al. 2022. “The Impacts of the Current Drought on Wildlife in Kenya.” Wildlife Research and Training Institute (WRTI).

[ece370632-bib-0061] Moss, J. B. , and G. M. While . 2021. “The Thermal Environment as a Moderator of Social Evolution.” Biological Reviews 96, no. 6: 2890–2910. 34309173 10.1111/brv.12784

[ece370632-bib-0063] National Drought Management Authority . 2022. “Laikipia County Drought Early Warning Bulletin for December 2022.”

[ece370632-bib-0064] Nicholson, A. 1954. “An Outline of the Dynamics of Animal Populations.” Australian Journal of Zoology 2: 9–65.

[ece370632-bib-0065] Nuñez, C. M. V. , J. S. Adelman , and D. I. Rubenstein . 2015. “Sociality Increases Juvenile Survival After a Catastrophic Event in the Feral Horse ( *Equus caballus* ).” Behavioral Ecology 26: 138–147.

[ece370632-bib-0066] Ogutu, J. O. , and N. Owen‐Smith . 2003. “ENSO, Rainfall and Temperature Influences on Extreme Population Declines Among African Savanna Ungulates.” Ecology Letters 6: 412–419.

[ece370632-bib-0067] Ostwald, M. M. , C. R. da Silva , and K. C. Seltmann . 2024. “How Does Climate Change Impact Social Bees and Bee Sociality?” Journal of Animal Ecology 93: 1610–1621. 39101348 10.1111/1365-2656.14160

[ece370632-bib-0068] Quinlan, R. J. 2007. “Human Parental Effort and Environmental Risk.” Proceedings of the Royal Society B: Biological Sciences 274: 121–125. 10.1098/rspb.2006.3690 PMC1679876 17134996

[ece370632-bib-0069] Ramona, R. , T. H. Clutton‐Brock , and M. B. Manser . 2019. “Drought Decreases Cooperative Sentinel Behavior and Affects Vocal Coordination in Meerkats.” Behavioral Ecology 30: 1558–1566.

[ece370632-bib-0070] Ramp, C. , W. Hagen , P. Palsbøll , M. Bérubé , and R. Sears . 2010. “Age‐Related Multi‐Year Associations in Female Humpback Whales ( *Megaptera novaeangliae* ).” Behavioral Ecology and Sociobiology 64: 1563–1576.

[ece370632-bib-0071] Raviv, L. , S. L. Jacobson , J. M. Plotnik , J. Bowman , V. Lynch , and A. Benítez‐Burraco . 2023. “Elephants as an Animal Model for Self‐Domestication.” Proceedings of the National Academy of Sciences of the United States of America 120: e2208607120. 37011191 10.1073/pnas.2208607120 PMC10104499

[ece370632-bib-0072] Redfern, J. V. , R. Grant , H. Biggs , and W. M. Getz . 2003. “Surface‐Water Constraints on Herbivore Foraging in the Kruger National Park, South Africa.” Ecology 84: 2092–2107.

[ece370632-bib-0073] Rondeau, S. , and N. E. Raine . 2024. “Unveiling the Submerged Secrets: Bumblebee Queens' Resilience to Flooding.” Biology Letters 20, no. 4: 20230609. 38626803 10.1098/rsbl.2023.0609 PMC11022157

[ece370632-bib-0074] Rubenstein, D. I. 1993. “The Ecology of Female Social Behavior in Horses, Zebras, and Asses.” In Animal Societies: Individuals, Interactions, and Organization, edited by P. J. Jarman and A. Rossiter , 13–28. Kyoto, Japan: Kyoto University.

[ece370632-bib-0075] Rubenstein, D. I. 2010. “Ecology, Social Behavior, and Conservation in Zebras.” In Advances in the Study of Behavior, 231–258. Oxford, UK: Elsevier Press.

[ece370632-bib-0076] Rubenstein, D. I. , and M. Hack . 2004. “Natural and Sexual Selection and the Evolution of Multi‐Level Societies: Insights From Zebras With Comparisons to Primates.” In Sexual Selection in Primates: New and Comparative Perspectives, edited by P. M. Kappeler and C. P. van Schaik , 266–279. New York: Cambridge University Press.

[ece370632-bib-0077] Rubenstein, D. I. , and C. M. Nuñez . 2009. “Sociality and Reproductive Skew in Horses and Zebras.” In Reproductive Skew in Vertebrates: Proximate and Ultimate Causes, edited by R. Hager and C. B. Jones , 196–226. Cambridge, UK: Cambridge University Press.

[ece370632-bib-0078] Rubenstein, D. R. , and I. J. Lovette . 2007. “Temporal Environmental Variability Drives the Evolution of Cooperative Breeding in Birds.” Current Biology 17: 1414–1419. 17702577 10.1016/j.cub.2007.07.032

[ece370632-bib-0079] Rymer, T. L. , N. Pillay , and C. Schradin . 2016. “Resilience to Droughts in Mammals: A Conceptual Framework for Estimating Vulnerability of a Single Species.” Quarterly Review of Biology 91: 133–176. 27405222 10.1086/686810

[ece370632-bib-0081] Salinas Ruíz, J. , O. A. Montesinos López , G. Hernández Ramírez , and J. Crossa Hiriart . 2023. “Generalized Linear Mixed Models for Proportions and Percentages.” In Generalized Linear Mixed Models With Applications in Agriculture and Biology, 209–278. Cham, Switzerland: Springer International Publishing.

[ece370632-bib-0082] Silk, J. B. , S. C. Alberts , and J. Altmann . 2003. “Social Bonds of Female Baboons Enhance Infant Survival.” Science 302: 1231–1234. 14615543 10.1126/science.1088580

[ece370632-bib-0083] Silk, J. B. , J. C. Beehner , T. J. Bergman , et al. 2009. “The Benefits of Social Capital: Close Social Bonds Among Female Baboons Enhance Offspring Survival.” Proceedings of the Royal Society B: Biological Sciences 276: 3099–3104. 10.1098/rspb.2009.0681 PMC2817129 19515668

[ece370632-bib-0084] Silk, J. B. , J. C. Beehner , T. J. Bergman , et al. 2010. “Strong and Consistent Social Bonds Enhance the Longevity of Female Baboons.” Current Biology 20: 1359–1361. 20598541 10.1016/j.cub.2010.05.067

[ece370632-bib-0085] Silva, S. , A. D. G. Rangeewa , and S. Kryazhimskiy . 2011. “The Dynamics of Social Networks in Asian Elephants.” BMC Ecology 11: 1–15. 21794147 10.1186/1472-6785-11-17 PMC3199741

[ece370632-bib-0086] Simpson, H. I. , S. A. Rands , and C. J. Nicol . 2012. “Social Structure, Vigilance and Behaviour of Plains Zebra ( *Equus burchellii* ): A 5‐Year Case Study of Individuals Living on a Managed Wildlife Reserve.” Acta Theriologica 57: 111–120.

[ece370632-bib-0087] Snyder‐Mackler, N. , J. R. Burger , L. Gaydosh , et al. 2020. “Social Determinants of Health and Survival in Humans and Other Animals.” Science 368: eaax9553. 32439765 10.1126/science.aax9553 PMC7398600

[ece370632-bib-0089] Testard, C. , S. M. Larson , M. M. Watowich , et al. 2021. “Rhesus Macaques Build New Social Connections After a Natural Disaster.” Current Biology 31: 2299–2309.e7. 33836140 10.1016/j.cub.2021.03.029 PMC8187277

[ece370632-bib-0090] Tong, W. , B. Shapiro , and D. I. Rubenstein . 2015. “Genetic Relatedness in Two‐Tiered Plains Zebra Societies Suggests That Females Choose to Associate With Kin.” Behaviour 152: 2059–2078.

[ece370632-bib-0091] Trivers, R. L. 1974. “Parent‐Offspring Conflict.” American Zoologist 14: 249–264.

[ece370632-bib-0092] Varpe, Ø. 2017. “Life History Adaptations to Seasonality.” Integrative and Comparative Biology 57: 943–960. 29045732 10.1093/icb/icx123

[ece370632-bib-0093] Waterman, J. M. , and M. B. Fenton . 2000. “The Effect of Drought on the Social Structure and Use of Space in Cape Ground Squirrels, *Xerus inauris* .” Ecoscience 7: 131–136.

[ece370632-bib-0095] Xie, B. , V. Daunay , T. C. Petersen , and E. F. Briefer . 2024. “Vocal Repertoire and Individuality in the Plains Zebra ( *Equus quagga* ).” Royal Society Open Science 11, no. 7: 240477. 39076369 10.1098/rsos.240477 PMC11286140

