# Supplementary material for: Plains Zebras Prioritize Foraging Without Sacrificing Social Bonds During a Severe Drought
Source: Ecol Evol. 2025 Jan 8;15(1):e70632. doi: 10.1002/ece3.70632 (PMC11710937; doi:10.1002/ece3.70632)
Supplement: Supplementary file 1 — Data S1. [file ECE3-15-e70632-s001.docx]

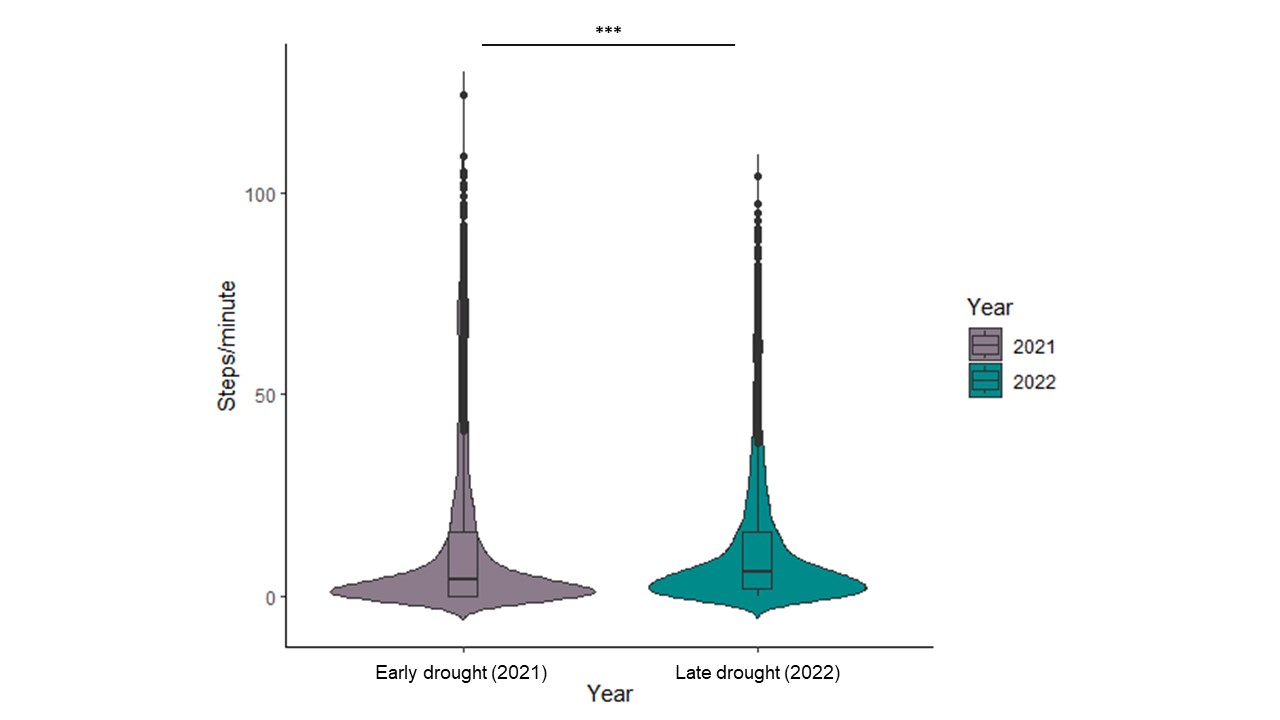


Fig. S1. Violin plot showing the steps per minute for the pre- (2021) and late (2022) drought periods.

*** < 0.001


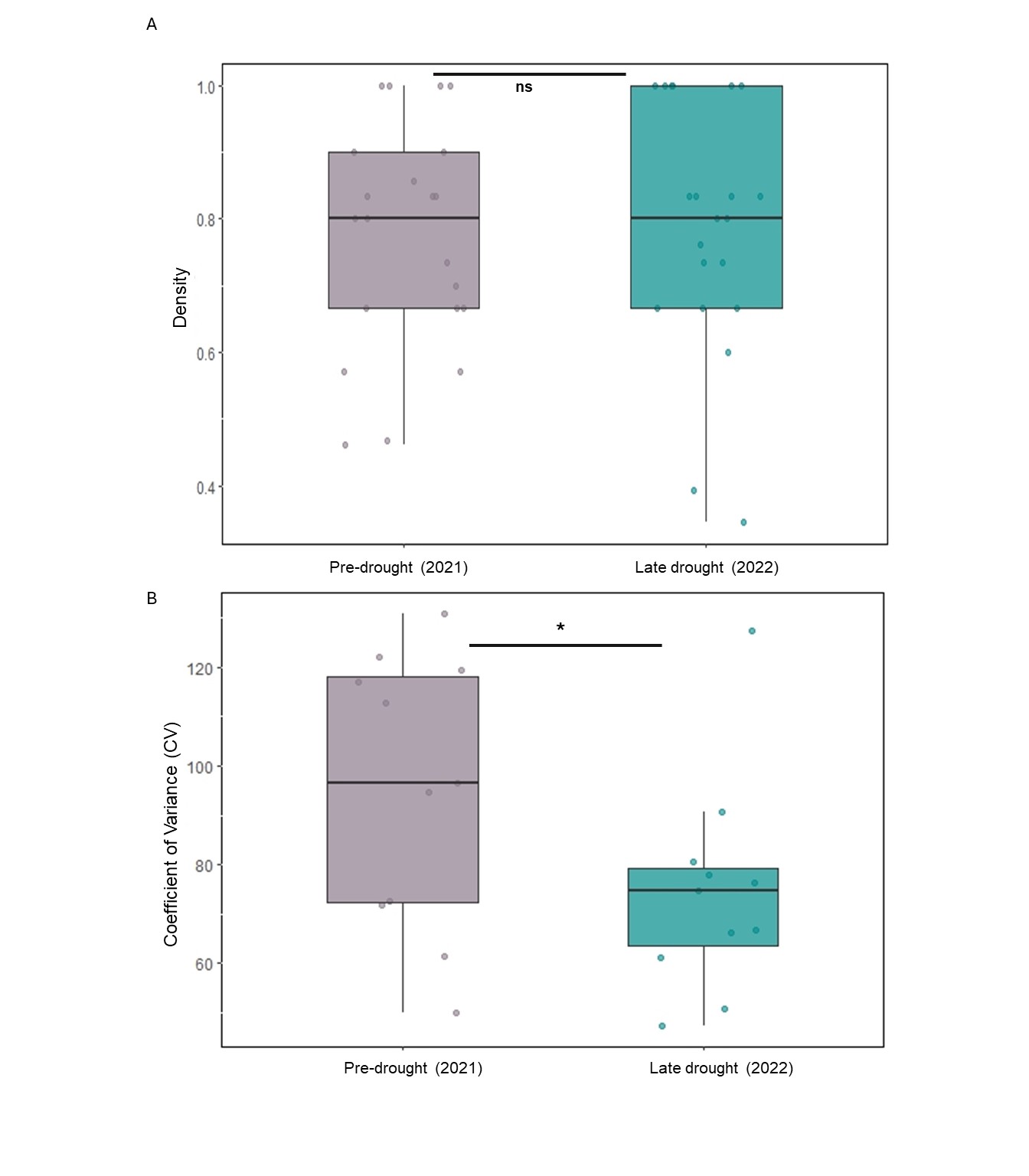


Fig. S2. Harem level changes in social connectivity across years in (*A*) harem network density plots for the pre- (2021) and late (2022) drought periods, (*B*) harem level coefficient of variance (CV) of association strength (SRI) for the pre- (2021) and late (2022) drought periods.

* < 0.05


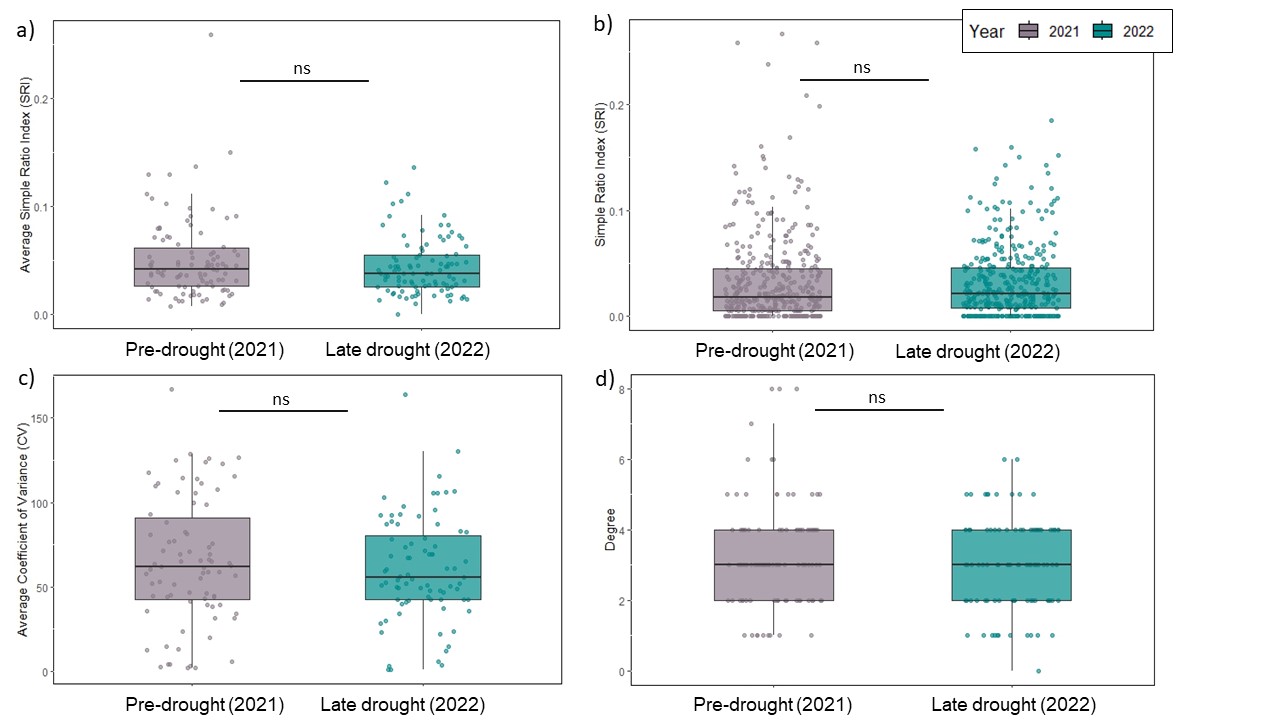


Fig. S3. Changes across the pre- (2021) to late (2022) drought periods in (*A*) each individual’s average SRI score, (*B*) dyadic SRI scores, (*C*) the coefficient of variance (CV) of each individual’s SRI scores, (*D*) degree of each individual.


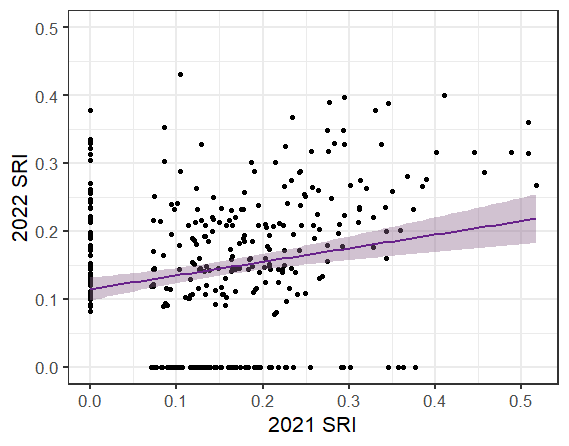


Fig. S4. Results of linear regression showing relationship between dyadic SRI in the pre- (2021) and late (2022) drought periods. Strength of social bond in 2021 predict strength of bond in 2022.

Table S1. Summary of the harems included in the sample and causes of composition/size change, when relevant.

*The stallion of MI6 disappeared pre- in 2021, and the two females of his harem joined the Designers, where they remained for the duration of the study period.

| Harem ID | Harem Size 2021 | Harem Size 2022 | Cause of composition change |
| --- | --- | --- | --- |
| Aristocrats (ARI) | 6 | 6 | -- |
| Constellations (CON) | 6 | 6 | -- |
| Designers (DE) | 6 | 6 | -- |
| Darksuns (DS) | 3 | 3 | -- |
| Fae (FAE) | 7 | 7 | -- |
| Hotrods (HOT) | 7 | 7 | -- |
| House of Usher (HU) | 3 | 4 | Immigration, adult female (dispersed from QDM) |
| Jedi Council (JC) | 5 | 4 | Juvenile male dispersal |
| Masters (MEAS) | 4 | 4 | -- |
| Maua (MAU) | 4 | 2 | Adult female death + juvenile male dispersal |
| Menagerie (MEN) | 5 | 6 | Recruitment |
| Mer’naar (MN) | 2 | 2 | -- |
| * MI6 | 3 | -- | * |
| Muses (MU) | 4 | 4 | -- |
| Quendi (QD) | 4 | 4 | -- |
| Queen’s Domain (QDM) | 13 | 11 | Juvenile female dispersal (to HU) |
| Saturnians (SAT) | 4 | 4 | -- |
| Skylords (SL) | 3 | 3 | -- |
| Spirits (SPI) | 5 | 5 | -- |
| Shards of the Flame (SoF) | 7 | 9 | Recruitment |
| Valkyries (VAL) | 4 | 3 | Adult female death |
| Zodiacs (ZO) | 4 | 3 | Adult female death |
| Total (N) | 109 | 103 |  |

# Table S2: Sample sizes of each age/sex class represented in the study.

| *The stallion of the former harem MI6 was present for one series of focal follows in 2021, and so his interactions were included. | | |
| --- | --- | --- |
| Age/sex class | 2021 Sample Size (N) | 2022 Sample Size (N) |
| Activity budget | | |
| Juvenile | 9 | 10 |
| Lactating Female | 8 | 9 |
| Nonlactating Female | 75 | 65 |
| Stallion | 21 | 21 |
| Interactions | | |
| Juvenile | 9 | 10 |
| Lactating Female | 8 | 11 |
| Nonlactating Female | 77 | 71 |
| Stallion | 22 * | 21 |

Table S3. Observations by individual across pre- (2021) and post (2022) drought.

Only individuals who were present across both years are included. Individuals were observed 14.42 ± 21.12 more times in 2021 than in 2022.

| **Harem ID** | **Individual ID** | **Focal Follows (2021)** | **Nearest Neighbor Sightings (2021)** | **Total Observations (2021)** | **Focal Follows (2022)** | **Nearest Neighbor Sightings (2022)** | **Total Observations (2022)** |
| --- | --- | --- | --- | --- | --- | --- | --- |
| ARI | Aethelwulf | 42 | 3 | **45** | 20 | 2 | **22** |
|  | Antoinette | 34 | 3 | **37** | 22 | 1 | **23** |
|  | Elanor | 44 | 0 | **44** | 21 | 2 | **23** |
|  | Isabella | 38 | 7 | **45** | 14 | 3 | **17** |
|  | Matilda | 39 | 7 | **46** | 21 | 1 | **22** |
|  | Victoria | 44 | 0 | **44** | 19 | 0 | **19** |
| CON | Andromeda | 54 | 6 | **60** | 34 | 4 | **38** |
|  | Carina | 44 | 13 | **57** | 31 | 5 | **36** |
|  | Cassiopeia | 50 | 4 | **54** | 28 | 4 | **32** |
|  | Cetus | 57 | 7 | **64** | 29 | 3 | **32** |
|  | Desdemona | 48 | 4 | **52** | 27 | 2 | **29** |
| DE | Juliet | 47 | 14 | **61** | 26 | 4 | **30** |
|  | Alexander | 32 | 0 | **32** | 38 | 1 | **39** |
|  | Jinx | 24 | 10 | **34** | 29 | 0 | **29** |
|  | Lyra | 16 | 5 | **21** | 39 | 0 | **39** |
|  | Prada | 31 | 8 | **39** | 38 | 2 | **40** |
|  | Vera | 27 | 3 | **30** | 37 | 1 | **38** |
|  | Vesper | 24 | 7 | **31** | 34 | 5 | **39** |
| DS | Arasne | 52 | 15 | **67** | 45 | 0 | **45** |
|  | Hespra | 55 | 12 | **67** | 30 | 0 | **30** |
|  | Merius | 55 | 3 | **58** | 44 | 2 | **46** |
| FAE | Banshee | 32 | 2 | **34** | 39 | 0 | **39** |
|  | Gawain | 24 | 3 | **27** | 36 | 3 | **39** |
|  | Harpy | 26 | 1 | **27** | 29 | 3 | **32** |
|  | Llorona | 26 | 2 | **28** | 28 | 6 | **34** |
|  | Naga | 26 | 4 | **30** | 29 | 2 | **31** |
|  | Selkie | 34 | 5 | **39** | 37 | 0 | **37** |
|  | Siren | 26 | 3 | **29** | 29 | 0 | **29** |
| HOT | Bugatti | 56 | 5 | **61** | 39 | 3 | **42** |
|  | Cadillac | 46 | 0 | **46** | 44 | 3 | **47** |
|  | Ferrari | 49 | 9 | **58** | 44 | 9 | **53** |
|  | Lamborghini | 45 | 9 | **54** | 40 | 3 | **43** |
|  | Maserati | 47 | 3 | **50** | 43 | 4 | **47** |
|  | Mercedes | 49 | 1 | **50** | 43 | 3 | **46** |
|  | Royce | 50 | 1 | **51** | 44 | 0 | **44** |
| HU | Lenoire | 45 | 17 | **62** | 60 | 9 | **69** |
|  | Sybil | 55 | 6 | **61** | 54 | 4 | **58** |
|  | Van Helsing | 45 | 3 | **48** | 53 | 2 | **55** |
| JC | Ahsoka | 50 | 8 | **58** | 49 | 2 | **51** |
|  | Depa | 52 | 12 | **64** | 54 | 3 | **57** |
|  | Luminara | 61 | 8 | **69** | 47 | 10 | **57** |
|  | Morian | 57 | 5 | **62** | 39 | 1 | **40** |
| MAS | Lorelei | 52 | 12 | **64** | 74 | 7 | **81** |
|  | Lucamine | 41 | 9 | **50** | 54 | 18 | **72** |
|  | Nessa | 39 | 2 | **41** | 36 | 1 | **37** |
|  | Raihan | 56 | 0 | **56** | 51 | 0 | **51** |
| MAU | Narcissus | 47 | 0 | **47** | 29 | 4 | **33** |
|  | Rose | 40 | 7 | **47** | 32 | 3 | **35** |
| MEN | Bayonetta | 37 | 4 | **41** | 63 | 8 | **71** |
|  | Sheena | 37 | 11 | **48** | 55 | 10 | **65** |
|  | Symmone | 35 | 10 | **45** | 60 | 6 | **66** |
|  | Velvet | 37 | 1 | **38** | 52 | 4 | **56** |
|  | Zaveid | 40 | 5 | **45** | 57 | 6 | **63** |
| MU | Apollo | 45 | 10 | **55** | 52 | 2 | **54** |
|  | Calliope | 43 | 22 | **65** | 51 | 12 | **63** |
|  | Khairon | 44 | 3 | **47** | 53 | 6 | **59** |
|  | Melete | 41 | 15 | **56** | 50 | 9 | **59** |
| QD | Galadriel | 47 | 15 | **62** | 36 | 3 | **39** |
|  | Nimrondel | 46 | 9 | **55** | 18 | 6 | **24** |
|  | Thranduil | 61 | 0 | **61** | 34 | 0 | **34** |
|  | Urania | 43 | 7 | **50** | 36 | 1 | **37** |
| QDM | Aminatu | 66 | 6 | **72** | 17 | 0 | **17** |
|  | Aomina | 64 | 9 | **73** | 43 | 1 | **44** |
|  | Beatrice | 66 | 8 | **74** | 24 | 4 | **28** |
|  | Hara | 65 | 5 | **70** | 24 | 0 | **24** |
|  | Himuku | 66 | 11 | **77** | 24 | 3 | **27** |
|  | Imogen | 66 | 2 | **68** | 24 | 4 | **28** |
|  | Kandake | 58 | 5 | **63** | 24 | 3 | **27** |
|  | Seraphina | 66 | 7 | **73** | 24 | 0 | **24** |
|  | Shinta | 69 | 7 | **76** | 23 | 3 | **26** |
|  | Tor | 66 | 5 | **71** | 24 | 4 | **28** |
|  | Yoshine | 66 | 1 | **67** | 24 | 3 | **27** |
|  | Zaki | 57 | 3 | **60** | 17 | 4 | **21** |
| SAT | Circe | 51 | 8 | **59** | 54 | 1 | **55** |
|  | Dione | 51 | 15 | **66** | 33 | 2 | **35** |
|  | Io | 52 | 8 | **60** | 33 | 2 | **35** |
|  | Zeus | 57 | 4 | **61** | 36 | 3 | **39** |
| SL | Aurithane | 53 | 3 | **56** | 48 | 1 | **49** |
|  | Endaria | 48 | 8 | **56** | 44 | 9 | **53** |
|  | Svala | 27 | 1 | **28** | 56 | 3 | **59** |
| SoF | Jehui | 57 | 7 | **64** | 19 | 0 | **19** |
|  | Kaalytz | 71 | 5 | **76** | 19 | 0 | **19** |
|  | Khav | 63 | 9 | **72** | 19 | 0 | **19** |
|  | Maaryvis | 69 | 5 | **74** | 19 | 0 | **19** |
|  | Revadkha | 67 | 3 | **70** | 15 | 3 | **18** |
|  | Vetaagh | 69 | 10 | **79** | 19 | 1 | **20** |
|  | Vheskharya | 68 | 5 | **73** | 15 | 0 | **15** |
| SPI | Absynthe | 38 | 18 | **56** | 26 | 5 | **31** |
|  | Amaretto | 40 | 8 | **48** | 27 | 8 | **35** |
|  | Champagne | 40 | 8 | **48** | 33 | 6 | **39** |
|  | Lemoncello | 34 | 4 | **38** | 36 | 6 | **42** |
|  | Mnazi | 40 | 4 | **44** | 28 | 0 | **28** |
| VAL | Baldur | 37 | 3 | **40** | 49 | 1 | **50** |
|  | Hella | 34 | 8 | **42** | 48 | 6 | **54** |
|  | Indun | 47 | 4 | **51** | 58 | 7 | **65** |
| ZO | Aquaria | 76 | 12 | **88** | 49 | 10 | **59** |
|  | Scorpio | 76 | 20 | **96** | 61 | 13 | **74** |
|  | Virgo | 56 | 21 | **77** | 47 | 6 | **53** |

Table S4. Ethogram of visual, acoustic, tactile, and chemical behaviors coded.

| Category | Component | Modality | Operational definition |
| --- | --- | --- | --- |
| Ears | Ears backwards | Visual | Pinnae backward, ears in a V shape |
|  | Ears pricked | Visual | Pinnae forward, tips up, ears moved closer together through activation of ear abductor |
|  | Ears different directions | Visual | Pinnae pointing in two different directions |
|  | Ears flat | Visual | Pinnae pointing down, tips pointing backwards, ears pressed flat against neck |
|  | Ears sideways | Visual | Pinnae pointing sideways from head, tips above the horizontal |
|  | Ears forward | Visual | Pinnae forward, ears in V shape |
|  | Ears flop | Visual | Pinnae down, tips horizontal from sides of head |
| Eyes | Inner brow raised | Visual | Angle of upper lid becomes more angular |
|  | Eyes open | Visual | Eyes open in a neutral state |
|  | Eyes half closed | Visual | Eyes opened halfway, eyelashes visible |
|  | Eyes closed | Visual | Eyes closed, eyelashes visible, no blinking present |
|  | Eyes roll | Visual | White of eye becomes visible |
| Upper lip | Upper lip normal | Visual | Upper lip in default state |
|  | Upper lip pointing | Visual | Upper lip extended beyond lower lip |
|  | Upper lip lifted | Visual | Upper lip lifted/curled to create wrinkles on the surface; teeth may be visible |
| Neck position | Neck horizontal | Visual | Neck held at a horizontal angle from the shoulder, parallel with the ground |
|  | Neck normal | Visual | Neck neutral, at approximately a 45-degree angle from the ground |
|  | Neck high | Visual | Neck held above 45 degrees |
|  | Neck low | Visual | Neck held below horizontal |
|  | Neck backwards | Visual | Neck turned so head is pointing backwards toward tail |
|  | Neck stretched horizontal | Visual | Neck held horizontal and head outstretched to create a single line from tip of nose to withers |
|  | Neck sideways | Visual | Neck turned so head is pointing sideways |
|  | Neck stretched high | Visual | Neck held above 45 degrees and head outstretched to create a single line from tip of nose to withers |
| Tail | Tail flagging | Visual | Tail repeatedly moving in vertical plane |
|  | Tail switching | Visual | Tail repeatedly moving in horizontal plane |
|  | Tail still | Visual | Tail motionless |
|  | Tail lifted | Visual | Tail lifted upwards away from rump |
|  | Tail clamped | Visual | Tail held tight to rump and may be visible between legs |
| Corner of lips | Corner of lips normal | Visual | Corner of lips in neutral state |
|  | Corner of lips back | Visual | Corner of lips pulled back towards ears, creating a rounded, “c” shaped contour |
|  | Corner of lips up | Visual | Corner of lips pulled up towards bridge of nose, creating a sharp angled contour |
| Lower lip/jaw | Jaw closed | Visual | Jaw in neutral state |
|  | Chewing | Visual | Mouth closed with chewing motion of the jaw |
|  | Depressed lip | Visual | Lower lip pulled down; teeth visible |
|  | Snapping | Visual | Mouth opened and closed >2 times consecutively in a chewing motion |
|  | Jaw open | Visual | Mouth held open. If >2 consecutive open and closing motions, code as ‘snapping’ |
|  | Licking lips | Visual | Tongue visible extruding from between lips, mouth typically closed |
|  | Relaxed lip | Visual | Lower lip hanging loose; teeth not visible |
| Neck motion | Horizontal neck shake | Visual | Repeated shaking of the head in a horizontal plane |
|  | Headbob | Visual | Repeated vertical lifting and lowering of the head |
|  | Head toss | Visual | Single, vigorous vertical plane lift |
|  | Flex at poll | Visual | Neck bends at poll (just behind the ears) creating a more rounded neck contour |
|  | Pass head over | Visual | Pass head and neck over the head/neck of a social partner |
|  | Pass head under | Visual | Pass head and neck under the head/neck of a social partner |
| Foreleg | Foreleg stomp | Visual | Vertically lift and lower foreleg |
|  | Foreleg strike | Visual | Extend foreleg forward |
|  | Rear | Visual | Both forelegs lifted and tucked |
| Hindleg | Kick threat | Visual | One or both hind legs licked as though to kick, but with no extension at the hock and no contact |
|  | Hind leg lift | Visual | Hind leg lifted and lowered vertically |
| Vocalization | Chirp | Vocal | Elsewhere, “short squeal” or “high pitched squeal”, a short, high-pitched vocalization that may or may not possess frequency modulation |
|  | Whuffle | Vocal | Elsewhere “blow” or “long snort’, an extended, nonvocal sound produced by exhalation through the nostrils with loose lips |
|  | Quagga-quagga | Vocal | Elsewhere called the “bark” or “i-ha”, a which is a rhythmic, repetitive vocalization used for long-distance communication, either mono or bi-syllabic and produced in bouts |
|  | Quagga-quagga whine | Vocal | Similar to the quagga-quagga, but higher pitched or whine-like, softer, and monosyllabic |
|  | Snort | Vocal | A short, explosive nonvocal exhalation through nostrils produced in contexts of alarm |
| Tactile | Kick | Tactile | One or both hindlegs extend fully and make contact with opponent |
|  | Nose-to-nose | Tactile | Touching noses, may or may not be accompanied by mutual nose blowing |
|  | Rub head | Tactile | Side or bottom of head is rubbed against the rump, shoulder, back, or side of partner |
|  | Nose-to-genital | Tactile | Nose lowered to the joint of a partner’s hindleg |
|  | Push | Tactile | Pushing laterally against an opponent using neck, head, or body |
|  | Lick partner | Tactile | Contacting partner with tongue |
|  | Nose-to-other | Tactile | Nose pressed to a part of the recipient’s body other than the nose or genitals |
|  | Allogroom | Tactile | Using upper teeth to groom social partner, usually the withers, shoulder, haunches, or hind legs |
|  | Rest head | Tactile | Head resting on top of the withers, back, or rump of partner; OR press head against partner’s side or neck |
|  | Nose-to-genital-thrust | Tactile | Nose lowered to the joint of a partner’s hindleg, then the head is jerked upward repeatedly against the partner’s belly/hip joint |
|  | Bite | Tactile | Bites opponent with teeth, may or may not hold on |
|  | Touch with lips | Tactile | Touches partner with extended or moving lips |
|  | Mount | Tactile | Full upper body raised and resting on the back of a partner |
|  | Nip | Tactile | Uses lips, but not teeth, to wrap around some part of the opponent’s body in a swift contact |
|  | Bite ankles | Tactile | Biting the lower front or hind legs of partner |
|  | Neck push down | Tactile | Neck pressing down on partner’s back, accompanied by shifting in weight as pressure is placed on partner |
|  | Lean | Tactile | Press entire body against partner |
|  | Neck wrestle | Tactile | Neck tangling and pressing against opponent |
| Chemical | Defecate | Chemical | Produces feces |
|  | Olfactory investigation | Chemical | Sniffs urine/feces |
| Genital | Erection | Visual | Penis emerges from prepuce and either fully or partially erects |
| Position | Rump swing | Visual | Swings hindquarters toward opponent |
|  | Interpose | Visual | Moves to stand between two individuals |
|  | Pivot present | Visual | Turns so that hindquarters are facing social partner and turns head to interact with them over their shoulder |
|  | Parallel | Visual | Standing beside social partner facing the same direction |
|  | Weave under neck | Tactile | Pass entire body underneath partner’s neck |
|  | Reverse parallel | Visual | Standing beside social partner facing opposite directions |
|  | Chest-to-rump | Tactile | Stand with chest pressed against partner’s rump |
|  | Block | Visual | Stand perpendicularly in front of partner to stop their movement |
|  | Back toward | Visual | Back towards social partner |

Table S5. The average percent of the daily activity budget

with standard deviation, dedicated to each behavior. Results of Mann Whitney U shows the significance of time spent in the pre- (2021) and late (2022) drought periods. Bolded behaviors are significant, italicized is trending towards significance.

| Behavior | 2021 | 2022 | W | p |
| --- | --- | --- | --- | --- |
| Drinking | 1.7 ± 1.55% | 1.4 ± 1.63% | 15 | 0.8 |
| **Independent grazing** | **42.17 ± 21.8%** | **53.76 ± 22.22%** | **3908** | **< 0.001***** |
| Geophagy | 3.3 ± 3.29% | 3.2% | 2 | 1 |
| Elimination | 0.42 ± 0.244% | 0.51± 0.31% | 753 | 0.2535 |
| Olfactory inspection | 0.38 ± 0.44% | 0.47 ± 0.50% | 204 | 0.3762 |
| Travelling | 13.97 ± 10.56% | 12.1 ± 10.76% | 6936 | 0.1044 |
| **Vigilant** | **4.6 ± 5.77%** | **2.6 ± 2.46%** | **4995** | **0.0083 ***** |
| Resting | 12 ± 12.89% | 17 ± 18.1% | 1069 | 0.1685 |
| Hygiene | 1.7 ± 2.56% | 1.8 ± 2.76% | 5478 | 0.5103 |
| **Standing** | **9.8 ± 10.62%** | **7.1 ± 7.76%** | **6291** | **0.00893 ***** |
| *Other* | *2.10 ± 2.90%* | *17.00 ± 17.33%* | *8* | *0.05994* |
| Passive Socializing | 13.37 ± 14.19% | 12.56 ± 13.11% | 9693 | 0.5033 |
| Social grazing | 10.23 ± 9.35% | 12.57 ± 11.57% | 3958 | 0.4784 |
| **Social rest** | **19.01 ± 18.97%** | **12.53 ± 16.49%** | **1196** | **0.0536 *** |
| Active Socializing | 0.88 **±** 1.67% | 1.15 **±** 1.56% | 5816 | 0.3216 |
| Affiliation | 1.1 ± 2.3% | 1.4 ± 1.8% | 1327 | 0.316 |
| Aggression | 0.4 ± 0.55% | 0.24 ± 0.21% | 308 | 0.6602 |
| Greeting | 0.54 ± 0.47% | 1.1 ± 1.33% | 243 | 0.5314 |
| Harem maintenance | 1.2 ± 0.81% | 0.45 ± 0.062 | 23 | 0.1 |
| Sex | 1.8 ± 2.76% | 0% | -- | -- |
| Play | 4.9% | 3.2 ± 0.66% | 2 | 0.6667 |

Table S6. Coefficient of variance (CV) for the simple ratio indexes (SRI) within each harem with greater than 4 members.

Note that two harems (JC and MAS) reduced from 5 to 4 harem members, and thus were not included in the analysis for late drought.

* < 0.025, ** < 0.01, *** < 0.001

| Harem Name | Harem size (2021/2022) | 2021 (pre- drought) | | 2022 (late drought) | |
| --- | --- | --- | --- | --- | --- |
|  | | CV | p-value | CV | p-value |
| ARI | 6/6 | 72.68 | p = 0.17 | 50.92 | p = 0.75 |
| CON | 6/6 | 61.51 | p = 0.42 | 66.90 | p = 0.24 |
| DE | 6/6 | 112.68 | **p = 0.008 ***** | *80.60* | p = 0.09 |
| FAE | 7/7 | 122.00 | **p < 0.001 ***** | 107.44 | **p < 0.001 ***** |
| HOT | 7/7 | 117.05 | **p < 0.001 ***** | 76.46 | p = 0.06 |
| JC | 5/4 | 94.68 | **p = 0.024 *** | -- | -- |
| MAS | 5/4 | 96.67 | **p = 0.019 *** | -- | -- |
| MEN | 5/6 | 50.1 | p = 0.73 | 74.82 | p = 0.12 |
| QDM | 13/11 | 130.98 | **p < 0.001 ***** | 90.83 | **p = 0.002 ***** |
| SoF | 7/8 | 119.35 | **p < 0.001 ***** | 77.88 | p = 0.09 |
| SPI | 5/5 | 72.50 | p = 0.20 | 61.17 | p = 0.45 |

# Table S7. Average ± SD rate of interaction per thirty minutes by age/sex class

| **Age/sex class** | **Interactions/30 min** | |
| --- | --- | --- |
|  | pre- drought | late drought |
| Juvenile | 1.64 ± 3.00 | 0.727 ± 1.51 |
| Lactating | 3.58 ± 4.66 | 0.75 ± 1.53 |
| Nonlactating | 0.733 ± 1.49 | 0.264 ± 0.834 |
| Stallion | 1.41 ± 2.44 | 1.06 ± 2.05 |

Table S8. Multimodal signal components observed in 2021 and 2022. Color indicates the module in which the signal was classified. Red = Aggression, purple = Affiliation, blue = greeting/submission.

| ID | Nodes | Modality | Module membership 2021 | Module membership 2022 |
| --- | --- | --- | --- | --- |
| 1 | Ears backwards | Visual | 2 | 1 |
| 2 | Ears pricked | Visual | 1 | 2 |
| 3 | Ears different directions | Visual | 2 | 1 |
| 4 | Ears flat | Visual | 3 | 3 |
| 5 | Ears sideways | Visual | 1 | 1 |
| 6 | Ears forward | Visual | 1 | 2 |
| 7 | Ears flop | Visual | 1 | 1 |
| 8 | Inner brow raised | Visual | 3 | 2 |
| 9 | Eyes open | Visual | 1 | 1 |
| 10 | Eyes closed | Visual | 2 | 1 |
| 11 | Eyes half closed | Visual | 2 | 2 |
| 12 | Eyes roll | Visual | 3 |  |
| 13 | Upper lip normal | Visual | 1 | 1 |
| 14 | Upper lip pointing | Visual | 1 | 2 |
| 15 | Upper lip lifted | Visual | 3 | 2 |
| 16 | Neck horizontal | Visual | 1 | 1 |
| 17 | Neck normal | Visual | 1 | 1 |
| 18 | Neck high | Visual | 2 | 1 |
| 19 | Neck low | Visual | 3 | 2 |
| 20 | Neck backwards | Visual | 3 | 2 |
| 21 | Neck stretched horizontal | Visual | 3 | 2 |
| 22 | Neck sideways | Visual | 1 | 1 |
| 23 | Neck stretched high | Visual | 2 | 2 |
| 24 | Tail flagging | Visual | 3 | 3 |
| 25 | Tail switching | Visual | 1 | 1 |
| 26 | Tail still | Visual | 2 | 1 |
| 27 | Tail lifted | Visual | 3 | 2 |
| 28 | Tail clamped | Visual | 3 | 2 |
| 29 | Corner of lips normal | Visual | 1 | 1 |
| 30 | Corner of lips back | Visual | 3 | 2 |
| 31 | Corner of lips up | Visual | 3 | 2 |
| 32 | Jaw closed | Visual | 1 | 1 |
| 33 | Chewing | Visual | 1 | 1 |
| 34 | Depressed lip | Visual | 3 | 2 |
| 35 | Jaw open | Visual | 3 | 2 |
| 36 | Licking lips | Visual | 2 |  |
| 37 | Snapping | Visual | 3 | 2 |
| 38 | Relaxed lip | Visual | 2 | 1 |
| 39 | Horizontal neck shake | Visual | 1 | 2 |
| 40 | Headbob | Visual | 1 | 1 |
| 41 | Head toss | Visual | 3 | 3 |
| 42 | Flex at poll | Visual | 3 | 3 |
| 43 | Pass head over | Visual | 2 |  |
| 44 | Pass head under | Visual | 1 | 1 |
| 45 | Foreleg stomp | Visual | 3 | 2 |
| 46 | Kick threat | Visual | 3 | 3 |
| 47 | Hind leg lift | Visual | 3 |  |
| 48 | Chirp | Vocal | 3 | 3 |
| 49 | Whuffle | Vocal | 2 |  |
| 50 | Quagga-quagga | Vocal | 2 | 2 |
| 51 | Quagga-quagga whine | Vocal | 1 |  |
| 52 | Kick | Tactile | 3 | 3 |
| 53 | Nose-to-nose | Tactile | 3 | 2 |
| 54 | Rub head | Tactile | 1 | 1 |
| 55 | Nose-to-genital | Tactile | 1 | 1 |
| 56 | Push | Tactile | 1 | 2 |
| 57 | Allogroom | Tactile | 2 | 1 |
| 58 | Touch with lips | Tactile | 2 | 1 |
| 59 | Lick partner | Tactile | 2 | 2 |
| 60 | Bite | Tactile | 3 |  |
| 61 | Bite ankles | Tactile | 3 |  |
| 62 | Nose-to-other | Tactile | 2 | 2 |
| 63 | Rest head | Tactile | 2 | 1 |
| 64 | Nose-to-genital-thrust | Tactile | 1 | 1 |
| 65 | Nip | Tactile | 3 | 2 |
| 66 | Mount | Tactile | 1 | 1 |
| 67 | Neck push down | Tactile | 2 |  |
| 68 | Lean | Tactile | 1 |  |
| 69 | Neck wrestle | Tactile | 1 |  |
| 70 | Defecate | Chemical | 3 | 1 |
| 71 | Olfactory investigation | Chemical | 3 | 2 |
| 72 | Erection | Visual | 1 | 1 |
| 73 | Rump swing | Visual | 3 | 3 |
| 74 | Interpose | Visual | 3 | 1 |
| 75 | Reverse parallel | Visual | 2 | 1 |
| 76 | Parallel | Visual | 1 | 2 |
| 77 | Pivot present | Tactile | 1 |  |
| 78 | Weave under neck | Visual | 1 |  |
| 79 | Chest-to-rump | Tactile | 1 | 1 |
| 80 | Block | Visual | 2 | 2 |
| 81 | Back toward | Visual | 3 |  |

LMM Outputs S1. GLMM outputs for activity budgets

Active Socializing

|  | Estimate | Standard Error | z-value | Pr(>\|z\|) |
| --- | --- | --- | --- | --- |
| (Intercept) | -4.783952 | 0.118134 | -40.50 | <0.001*** |
| Year 2022 | -0.063234 | 0.160809 | -0.39 | 0.69415 |
| PhenotypeLactating | 0.312367 | 0.296615 | 1.05 | 0.29229 |
| PhenotypeStallion | 0.223861 | 0.158334 | 1.41 | 0.15741 |
| PhenotypeJuvenile | -0.002124 | 0.243439 | -0.01 | 0.99304 |
| Year2022: PhenotypeLactating | 0.099271 | 0.510388 | 0.19 | 0.84578 |
| Year2022: phenotype Stallion | 0.266108 | 0.259100 | 1.03 | 0.30440 |
| **Year2022: phenotypeJuvenile** | **1.048906** | **0.358791** | **2.92** | **0.00346 **** |


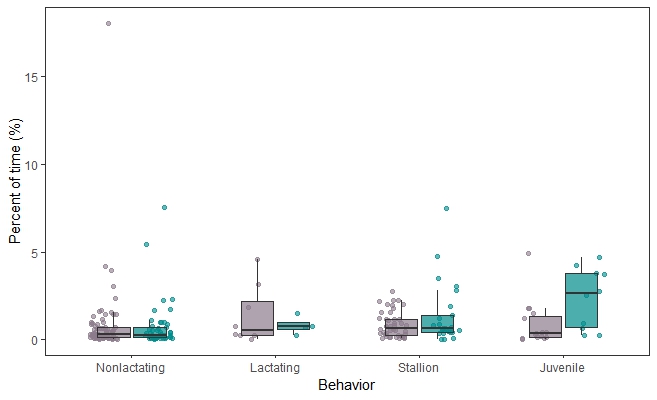


Aggression

|  | Estimate | Standard Error | z-value | Pr(>\|z\|) |
| --- | --- | --- | --- | --- |
| **(Intercept)** | -5.68995 | 0.18023 | -31.571 | **<0.001***** |
| Year 2022 | -0.18008 | 0.27447 | -0.656 | 0.5118 |
| PhenotypeLactating | -0.19004 | 0.48804 | -0.389 | 0.6970 |
| *PhenotypeStallion* | *0.57223* | *0.30572* | *1.872* | *0.0612 .* |
| PhenotypeJuvenile | -0.65132 | 0.54330 | -1.199 | 0.2306 |
| Year2022: phenotype Stallion | -0.07666 | 0.55876 | -0.137 | 0.8909 |


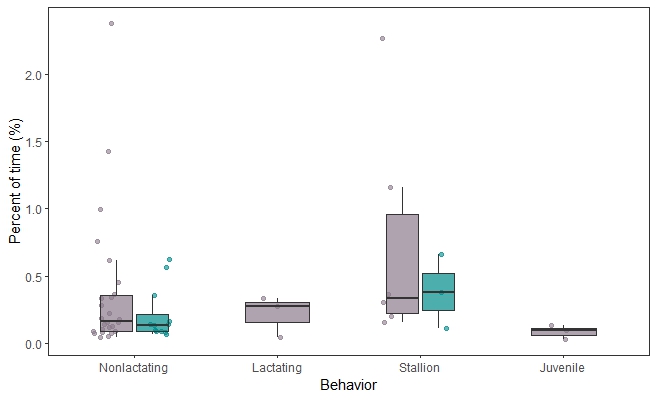


Affiliation

|  | Estimate | Standard Error | z-value | Pr(>\|z\|) |
| --- | --- | --- | --- | --- |
| **(Intercept)** | **-4.55757** | **0.17845** | **-25.540** | **<0.001***** |
| Year 2022 | 0.01106 | 0.21017 | 0.053 | 0.958 |
| PhenotypeLactating | 0.55832 | 0.36303 | 1.538 | 0.124 |
| PhenotypeStallion | -0.20284 | 0.27994 | -0.725 | 0.469 |
| PhenotypeJuvenile | 0.09710 | 0.33043 | 0.294 | 0.769 |
| Year2022: PhenotypeLactating | -0.41528 | 0.57907 | -0.717 | 0.473 |
| Year2022: phenotype Stallion | 0.27735 | 0.46186 | 0.601 | 0.548 |
| *Year2022: phenotypeJuvenile* | *0.78291* | *0.47459* | *1.650* | *0.099 .* |


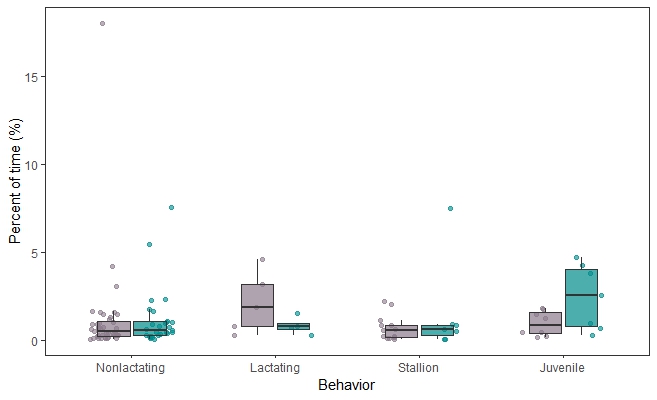


Elimination

|  | Estimate | Standard Error | z-value | Pr(>\|z\|) |
| --- | --- | --- | --- | --- |
| **(Intercept)** | **-5.61873** | **0.13864** | **-40.53** | **<0.001** |
| Year 2022 | 0.23444 | 0.15664 | 1.50 | 0.1345 |
| PhenotypeLactating | -0.01811 | 0.41585 | -0.04 | 0.9653 |
| *PhenotypeStallion* | *0.33208* | *0.17943* | *1.85* | *0.0642 .* |
| PhenotypeJuvenile | 0.10923 | 0.27974 | 0.39 | 0.6962 |
| Year2022: PhenotypeLactating | 0.22792 | 0.46154 | 0.49 | 0.6214 |
| Year2022: phenotype Stallion | -0.39689 | 0.25926 | -1.53 | 0.1258 |
| Year2022: phenotypeJuvenile | 0.26773 | 0.43873 | 0.61 | 0.5417 |


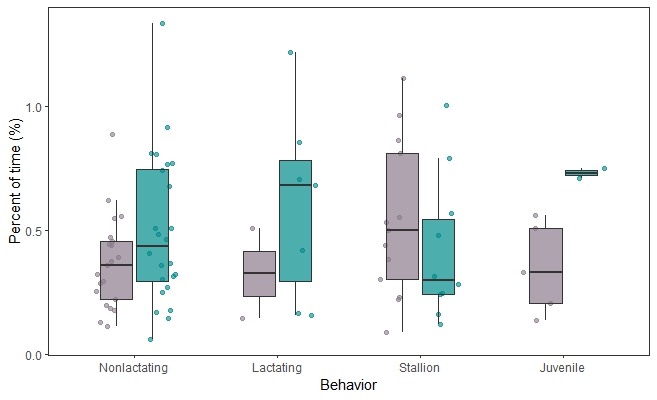


Greeting

|  | Estimate | Standard Error | z-value | Pr(>\|z\|) |
| --- | --- | --- | --- | --- |
| **(Intercept)** | **-5.4227** | **0.3504** | **-15.476** | **<0.001***** |
| Year 2022 | -0.3673 | 0.4327 | -0.849 | 0.3960 |
| PhenotypeStallion | 0.4863 | 0.3553 | 1.369 | 0.1711 |
| PhenotypeJuvenile | -0.1563 | 0.5335 | -0.293 | 0.7695 |
| **Year2022: phenotype Stallion** | **1.1638** | **0.4713** | **2.470** | **0.0135 *** |
| Year2022: phenotypeJuvenile | 0.5038 | 0.9896 | 0.509 | 0.6107 |


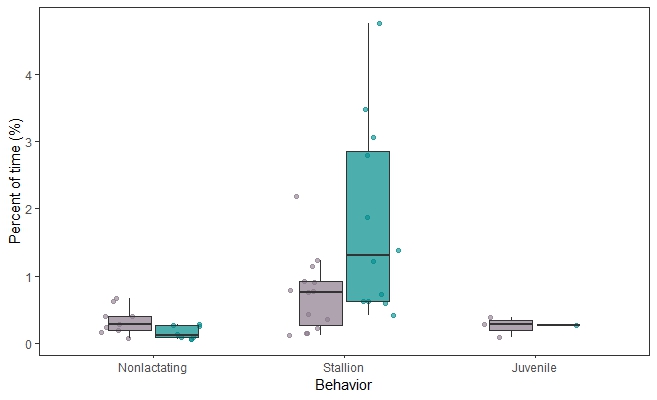


Independent grazing

|  | Estimate | Standard Error | z-value | Pr(>\|z\|) |
| --- | --- | --- | --- | --- |
| **(Intercept)** | **-0.3340** | **0.1217** | **-2.745** | **0.00605 **** |
| **Year 2022** | **0.4754** | **0.1464** | **3.247** | **0.00117 **** |
| PhenotypeLactating | -0.3702 | 0.3406 | -1.139 | 0.256288 |
| PhenotypeStallion | 0.2653 | 0.2136 | 1.289 | 0.199190 |
| PhenotypeJuvenile | -0.1314 | 0.3122 | -0.478 | 0.633328 |
| Year2022: PhenotypeLactating | 0.4986 | 0.4616 | 0.777 | 0.438111 |
| Year2022: phenotype Stallion | -0.1821 | 0.3026 | -0.620 | 0.536377 |
| Year2022: phenotypeJuvenile | -0.4945 | 0.5118 | -0.966 | 0.33396 |


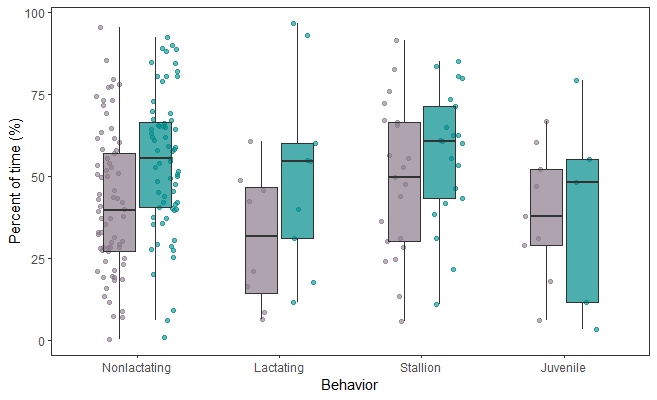


Olfactory Inspection

|  | Estimate | Standard Error | z-value | Pr(>\|z\|) |
| --- | --- | --- | --- | --- |
| **(Intercept)** | -5.69313 | 0.21419 | -26.580 | **<0.001***** |
| Year 2022 | 0.02411 | 0.31996 | 0.075 | 0.940 |
| PhenotypeLactating | 0.89675 | 0.59422 | 1.509 | 0.131 |
| PhenotypeStallion | 0.39559 | 0.30968 | 1.277 | 0.201 |
| PhenotypeJuvenile | -0.41639 | 0.52641 | -0.791 | 0.429 |
| Year2022: phenotype Stallion | 0.14590 | 0.45753 | 0.319 | 0.750 |
| Year2022: phenotypeJuvenile | 0.69381 | 0.90817 | 0.764 | 0.445 |


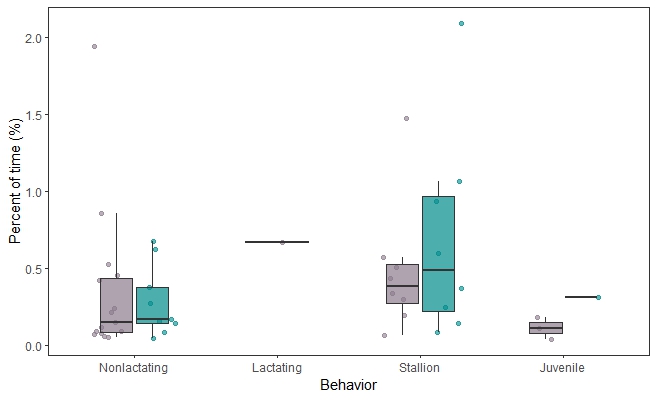


Passive Socializing

|  | Estimate | Standard Error | z-value | Pr(>\|z\|) |
| --- | --- | --- | --- | --- |
| **(Intercept)** | **-1.86031** | **0.11516** | **-16.154** | **<0.001***** |
| Year 2022 | 0.01256 | 0.12845 | -0.098 | 0.9221 |
| PhenotypeLactating | -0.13434 | 0.37634 | -0.357 | 0.7211 |
| *PhenotypeStallion* | *-0.37669* | *0.20624* | *-1.826* | *0.0678 .* |
| PhenotypeJuvenile | 0.15496 | 0.26605 | 0.582 | 0.5603 |
| Year2022: PhenotypeLactating | 0.29040 | 0.59422 | 0.489 | 0.6250 |
| Year2022: phenotype Stallion | 0.07382 | 0.30263 | 0.244 | 0.8073 |
| *Year2022: phenotypeJuvenile* | *-0.77686* | *0.41160* | *-1.887* | *0.0591 .* |


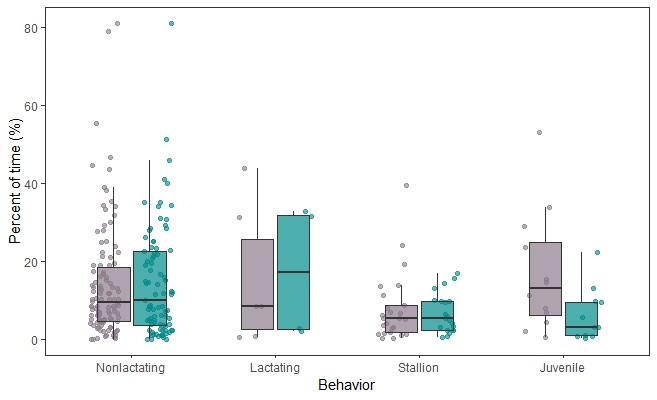


Resting

|  | Estimate | Standard Error | z-value | Pr(>\|z\|) |
| --- | --- | --- | --- | --- |
| **(Intercept)** | **-1.96849** | **0.18135** | **-10.855** | **<0.001***** |
| Year 2022 | -0.00955 | 0.21869 | -0.044 | 0.9652 |
| PhenotypeLactating | -1.06529 | 0.72259 | -1.474 | 0.1404 |
| PhenotypeStallion | -0.03129 | 0.28143 | -0.111 | 0.9115 |
| *PhenotypeJuvenile* | *1.47391* | *0.78227* | *1.884* | *0.0595 .* |
| Year2022: PhenotypeLactating | 0.61790 | 1.18987 | 0.519 | 0.6036 |
| Year2022: phenotype Stallion | 0.10322 | 0.40745 | 0.253 | 0.8000 |
| Year2022: phenotypeJuvenile | 0.21082 | 0.82123 | 0.257 | 0.7974 |


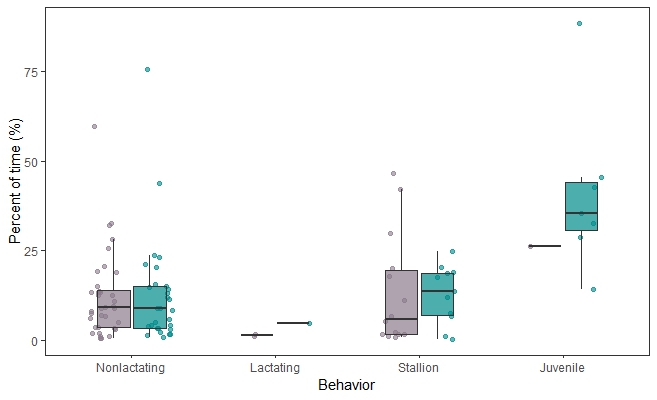


Hygiene

|  | Estimate | Standard Error | z-value | Pr(>\|z\|) |
| --- | --- | --- | --- | --- |
| **(Intercept)** | **-4.007556** | **0.106560** | **-37.61** | **<0.001***** |
| Year 2022 | -0.045459 | 0.141777 | -0.32 | 0.748 |
| PhenotypeLactating | -0.231264 | 0.359123 | -0.64 | 0.520 |
| PhenotypeStallion | -0.006835 | 0.208434 | -0.03 | 0.974 |
| PhenotypeJuvenile | 0.271359 | 0.267779 | 1.01 | 0.311 |
| Year2022: PhenotypeLactating | -0.213635 | 0.476980 | -0.45 | 0.654 |
| Year2022: phenotype Stallion | 0.091172 | 0.293372 | 0.31 | 0.756 |
| Year2022: phenotypeJuvenile | -0.121877 | 0.379431 | -0.32 | 0.748 |


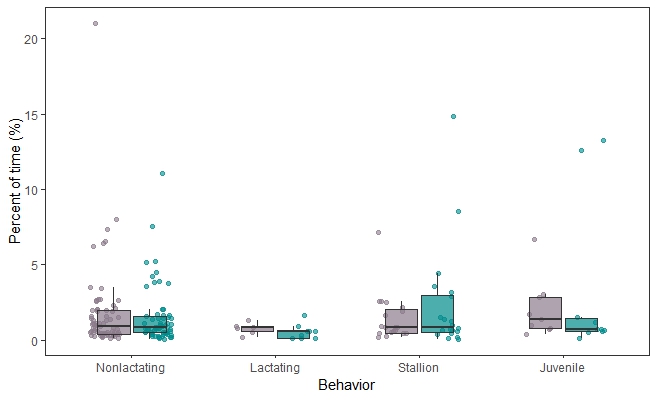


Social Grazing

|  | Estimate | Standard Error | z-value | Pr(>\|z\|) |
| --- | --- | --- | --- | --- |
| **(Intercept)** | **-2.18695** | **0.13396** | **-16.326** | **<0.001***** |
| **Year 2022** | **0.27943** | **0.13681** | **2.043** | **0.04110 *** |
| PhenotypeLactating | -0.65353 | 0.45455 | -1.438 | 0.15050 |
| *PhenotypeStallion* | *-0.40602* | *0.22030* | *-1.843* | *0.06532 .* |
| PhenotypeJuvenile | 0.25065 | 0.27930 | 0.897 | 0.36951 |
| Year2022: PhenotypeLactating | 0.80921 | 0.59926 | 1.350 | 0.17691 |
| Year2022: phenotype Stallion | -0.02615 | 0.31279 | -0.084 | 0.93336 |
| **Year2022: phenotypeJuvenile** | **-1.39587** | **0.44680** | **-3.124** | **0.00178 **** |


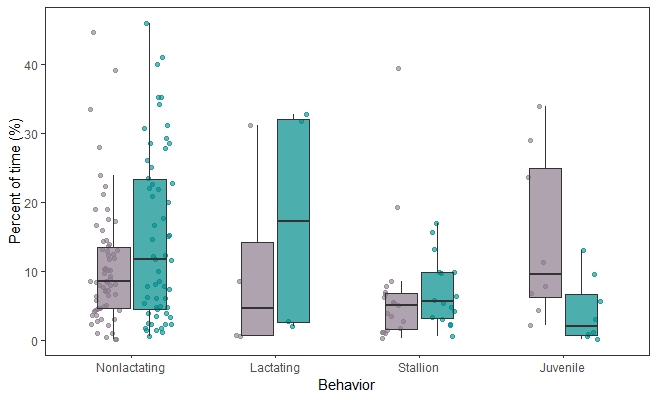


Social Rest

|  | Estimate | Standard Error | z-value | Pr(>\|z\|) |
| --- | --- | --- | --- | --- |
| **(Intercept)** | **-1.47383** | **0.18459** | **-7.984** | **<0.001***** |
| Year 2022 | -0.39239 | 0.24723 | -1.587 | 0.112 |
| PhenotypeLactating | 0.45502 | 0.69435 | 0.655 | 0.512 |
| PhenotypeStallion | -0.34558 | 0.43906 | -0.787 | 0.431 |
| PhenotypeJuvenile | -0.01062 | 0.52535 | -0.020 | 0.984 |
| Year2022: phenotype Stallion | 0.19007 | 0.66164 | 0.287 | 0.774 |
| Year2022: phenotype Juvenile | 0.20886 | 0.82013 | 0.255 | 0.799 |


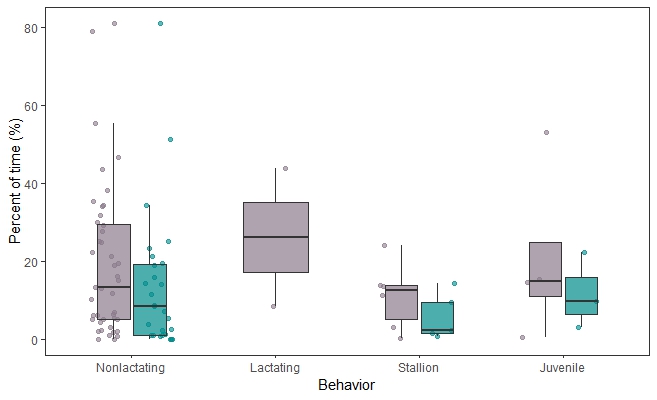


Standing

|  | Estimate | Standard Error | z-value | Pr(>\|z\|) |
| --- | --- | --- | --- | --- |
| **(Intercept)** | **-2.25984** | **0.10730** | **-21.060** | **<0.001***** |
| **Year 2022** | **-0.38494** | **0.15387** | **-2.502** | **0.0124 *** |
| PhenotypeLactating | -0.14894 | 0.34171 | -0.436 | 0.6629 |
| PhenotypeStallion | 0.22366 | 0.20991 | 1.065 | 0.2867 |
| PhenotypeJuvenile | 0.01113 | 0.29714 | 0.037 | 0.9701 |
| Year2022: PhenotypeLactating | -0.06542 | 0.50263 | -0.130 | 0.8964 |
| Year2022: phenotype Stallion | 0.20681 | 0.30156 | 0.686 | 0.4928 |
| Year2022: phenotypeJuvenile | 0.44129 | 0.41384 | 1.066 | 0.2863 |


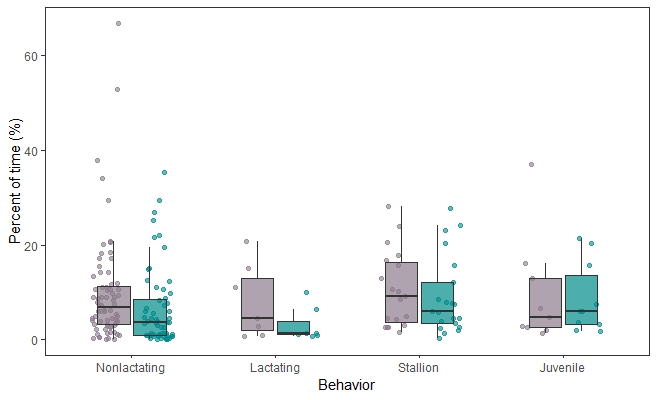


Travelling

|  | Estimate | Standard Error | z-value | Pr(>\|z\|) |
| --- | --- | --- | --- | --- |
| **(Intercept)** | **-1.87086** | **0.10729** | **-17.437** | **<0.001***** |
| Year 2022 | -0.16742 | 0.12548 | -1.334 | 0.182 |
| PhenotypeLactating | -0.31362 | 0.29006 | -1.081 | 0.280 |
| PhenotypeStallion | 0.04456 | 0.18047 | 0.247 | 0.805 |
| PhenotypeJuvenile | 0.35419 | 0.23956 | 1.478 | 0.139 |
| Year2022: PhenotypeLactating | 0.13809 | 0.41164 | 0.335 | 0.737 |
| Year2022: phenotype Stallion | 0.14037 | 0.25721 | 0.546 | 0.585 |
| Year2022: phenotypeJuvenile | -0.05514 | 0.34422 | -0.160 | 0.873 |


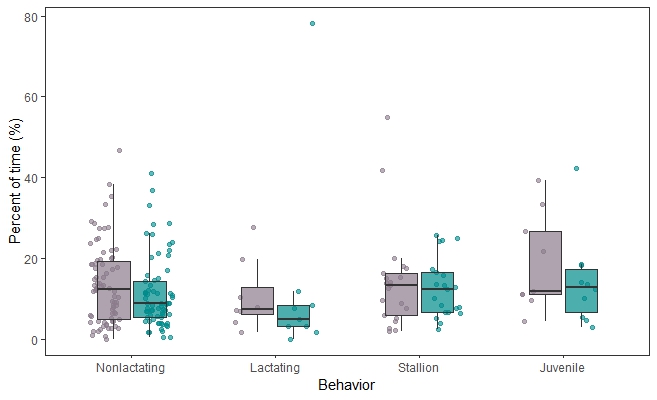


Vigilance

|  | Estimate | Standard Error | z-value | Pr(>\|z\|) |
| --- | --- | --- | --- | --- |
| **(Intercept)** | **-3.17500** | **0.10989** | **-28.893** | **<0.001***** |
| **Year 2022** | **-0.36300** | **0.16117** | **2.252** | **0.0243 *** |
| PhenotypeLactating | -0.61940 | 0.37733 | -1.642 | 0.1007 |
| PhenotypeStallion | 0.30197 | 0.18667 | 1.618 | 0.1057 |
| PhenotypeJuvenile | 0.19865 | 0.31438 | 0.632 | 0.5275 |
| Year2022: PhenotypeLactating | 0.57168 | 0.52462 | 1.090 | 0.2758 |
| Year2022: phenotype Stallion | 0.08383 | 0.27707 | 0.303 | 0.7622 |
| Year2022: phenotypeJuvenile | -0.25052 | 0.48471 | -0.517 | 0.6053 |


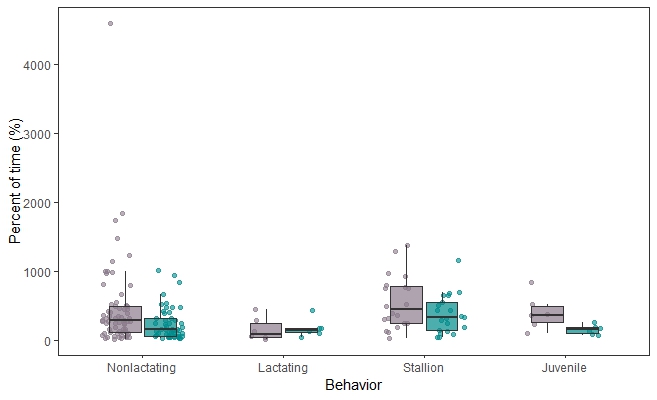


LMM Outputs S2: Steps per Minute LMM output

|  | Estimate | Standard Error | df | t-value | Pr(>\|t\|) |
| --- | --- | --- | --- | --- | --- |
| (Intercept) | 2.565e+00 | 3.828e-01 | 1.134e+00 | 6.70 | 0.0752. |
| **Year2022** | **3.466 e-01** | **5.391e-02** | **8.229 e+03** | **6.43** | **< 0.001 ***** |

Model selection based on AIC_c_

|  | K | AIC_c_ | Delta AIC_c_ | AIC_c_ Wt | Cum.Weight | Res.LL |
| --- | --- | --- | --- | --- | --- | --- |
| **Year only** | **6** | **38319.80** | **0.00** | **0.99** | **0.99** | **-19153.90** |
| Year + Phenotype | 9 | 38328.90 | 9.09 | 0.01 | 1.00 | -19155.44 |
| full | 12 | 38337.54 | 0.00 | 0.00 | 1.00 | -19156.71 |
| Random only | 5 | 38354.95 | 35.15 | 0.00 | 1.00 | -19172.47 |
| Phenotype only | 8 | 38362.88 | 43.08 | 0.00 | 1.00 | -19173.43 |

LMM Outputs S3. Interactions per 30 minutes LMM output

|  | Estimate | Standard Error | df | t-value | Pr(>\|t\|) |
| --- | --- | --- | --- | --- | --- |
| (Intercept) | -0.06585 | 0.11763 | 1.73905 | 0.560 | 0.63922 |
| **Year2022** | **-0,24153** | **0.07494** | **982.87530** | **-3.223** | **0.00121**** |
| **PhenotypeLactating** | **1.64699** | **0.23981** | **792.73690** | **6.868** | **<0.001***** |
| **PhenotypeStallion** | **0.37808** | **0.11316** | **170.33543** | **3.341** | **0.00103**** |
| **PhenotypeJuvenile** | **0.50944** | **0.17666** | **219.39717** | **2.884** | **0.00432**** |
| **Year2022:PhenotypeLactating** | **-1.35235** | **0.30852** | **839.92739** | **-4.383** | **<0.001***** |
| Year2022:PhenotypeStallion | 0.06145 | 0.14302 | 985.98210 | 0.430 | 0.66753 |
| Year2022:PhenotypeJuvenile | -0.27566 | 0.24921 | 569.50984 | -1.106 | 0.26914 |

Model selection based on AIC_c_

|  | K | AIC_c_ | Delta AIC_c_ | AIC_c_ Wt | Cum.Weight | Res.LL |
| --- | --- | --- | --- | --- | --- | --- |
| **Year X Phenotype** | **12** | **2667.41** | **0.00** | **1** | **1** | **-1376.05** |
| Year + Phenotype | 9 | 2787.61 | 11.20 | 0 | 0 | -1384.72 |
| Phenotype only | 8 | 2806.40 | 29.99 | 0 | 0 | -1395.13 |
| Year only | 6 | 2817.97 | 41.56 | 0 | 0 | -1402.94 |
| Random only | 5 | 2830.82 | 54.41 | 0 | 0 | -1410.38 |
